# A Survey on Free-Space Optical Communication with RF Backup: Models, Simulations, Experience, Machine Learning, Challenges and Future Directions

**DOI:** 10.3390/s25113310

**Published:** 2025-05-24

**Authors:** Sabai Phuchortham, Hakilo Sabit

**Affiliations:** School of Engineering, Computer and Mathematical Sciences, Auckland University of Technology, 6-24 St. Paul Street, Private Bag 92006, Auckland 1010, New Zealand; hakilo.sabit@aut.ac.nz

**Keywords:** free-space optical, radio frequency, hybrid FSO/RF, 5G, B5G, machine learning

## Abstract

As sensor technology integrates into modern life, diverse sensing devices have become essential for collecting critical data that enables human–machine interfaces such as autonomous vehicles and healthcare monitoring systems. However, the growing number of sensor devices places significant demands on network capacity, which is constrained by the limitations of radio frequency (RF) technology. RF-based communication faces challenges such as bandwidth congestion and interference in densely populated areas. To overcome these challenges, a combination of RF with free-space optical (FSO) communication is presented. FSO is a laser-based wireless solution that offers high data rates and secure communication, similar to fiber optics but without the need for physical cables. However, FSO is highly susceptible to atmospheric turbulence and conditions such as fog and smoke, which can degrade performance. By combining the strengths of both RF and FSO, a hybrid FSO/RF system can enhance network reliability, ensuring seamless communication in dynamic urban environments. This review examines hybrid FSO/RF systems, covering both theoretical models and real-world applications. Three categories of hybrid systems, namely hard switching, soft switching, and relay-based mechanisms, are proposed, with graphical models provided to improve understanding. In addition, multi-platform applications, including autonomous, unmanned aerial vehicles (UAVs), high-altitude platforms (HAPs), and satellites, are presented. Finally, the paper identifies key challenges and outlines future research directions for hybrid communication networks.

## 1. Introduction

Sensor technology forms the backbone of innovations in human–machine interfaces, smart cities, safety systems, the Internet of Things (IoT), and extended reality (XR). There are a variety of sensors—from temperature, heartbeat monitors, and wind speed detectors to chemical sensor devices. Those sensor applications are growing enormously, increasing opportunities to detect more advanced metrics [1]. Moreover, sensor technology combined with artificial intelligence (AI) technology, which relies on massive, high-quality, and real-time data, is increasingly applied in advanced sensor applications. As sensors continuously collect vast amounts of real-time data. A robust connectivity between these sensors and networks becomes essential [2]. This connectivity requires high speed and low latency to provide the best experience for applications. In particular, this requirement is crucial in time-sensitive applications such as autonomous vehicles, industrial automation, and healthcare monitoring systems where even millisecond delays can have significant consequences. Therefore, a backbone network is the core of the sensor infrastructure to support most data communication.

Initially, copper wire connections such as ADSL and VDSL were the foundation of the early backbone internet and sensor networks. Over time, this wired connection evolved into the passive optical network (PON), which applies fiber optics as a medium to transfer incredibly high speed and reliability over long distances. Eventually, fiber optic networks have become the backbone of data communication for modern high-speed internet and data services. However, as the demand for mobility and seamless connectivity grows, wired connections alone can no longer be sufficient. This shift from wired to wireless solutions has become essential. As a result, it led to the development of radio frequency (RF) wireless technologies such as LTE, 5G, satellite communication, microwave networks, and WiMAX, which offer flexibility and wide area coverage. RF has become the core of wireless communication that allows users to stay connected from virtually anywhere. Nevertheless, as the demand for mobile data grew continuously, RF systems encountered bandwidth capacity, faced congestion in densely populated [3], and limitations in specific environments, such as hospitals or radio-sensitive zones.

To address these RF challenges, free-space optical (FSO) communication is introduced. FSO transmits data using laser technology, similar to PON, but without requiring physical cables, often referred to as “fiberless” [4]. FSO offers high bandwidth, enhanced security, unregulated licenses, and immunity to electromagnetic interference. On the other hand, it requires a precise line-of-sight (LoS) alignment and is highly sensitive to atmospheric impairments such as fog, smoke, and atmospheric turbulence, which can drastically degrade signal quality. Fog and smoke primarily attenuate the optical beam through scattering, thereby reducing the received signal strength [5]. Besides, atmospheric turbulence induces random refractive-index fluctuations that distort the optical wavefront (phase) and cause intensity scintillations [6]. These turbulence-induced phase distortions lead to signal fading and are a major performance-limiting factor for FSO links, especially over long distances [7].

Since neither RF nor FSO can ensure reliable connectivity under all situations and weather conditions, a hybrid RF/FSO system offers a promising solution. The hybrid FSO/RF systems combine the high throughput of FSO links with the reliability of RF links during adverse climates. For example, NASA’s Laser Communications Relay Demonstration (LCRD) [8], Google’s Project Loon [9], Facebook’s Aquila [10], Terragraph Project [11], HAWK30 [12], DARPA’s FSO Experimental Network Experiment (FOENEX) [13], Thales Alenia Space Optical Communication [14], and Free-space experimental laser terminal (FELT II) [15]. In particular, this hybrid system is a strong candidate for beyond 5G (B5G) network [16]. These projects highlight the rising potential of hybrid FSO/RF systems to revolutionize not only the sensor networks but also transform the future of data transmission across global communication.

This survey explores hybrid FSO/RF articles from theoretical models to real-world implementations. Unlike previous reviews, this work makes a clear classification of hybrid systems with simple graphical models to facilitate understanding. We observed that there are three categories of hybrid systems, namely hard switching, soft switching, and relay-based mechanisms. To foster a simpler understanding of hybrid FSO/RF systems, we proposed graphical models of these categories. In addition, multi-platform applications, including autonomous unmanned aerial vehicles (UAVs), high-altitude platforms (HAPs), and satellites, are presented. Finally, the paper identifies key challenges and outlines future research directions for hybrid communication networks.

Hybrid FSO/RF systems have attracted significant research interest due to their potential. The system can address the limitations of traditional wireless networks, including bandwidth and security. This section provides a comprehensive review of relevant literature and outlines the unique contributions of this study.

Starting from early smoke signals to modern IP-based communication, the evolution of communication system history was presented by Raj and Majumder [17]. They also proposed a history of optical wireless communication (OWC) and introduced a hybrid OWC/RF system. The hybrid OWC/RF system can be separated into various categories. FSO is one of the subsections of OWC with other systems such as LiFi, VLC, and OCC systems. While RF can be separated into mmW, WiFi, and Bluetooth [18]. The potential of hybrid FSO/RF systems to mitigate propagation effects in various scenarios was highlighted by many studies [19,20,21,22,23,24]. While authors [25] try to update the hybrid system from 2015 to 2019. Also, a system a systematic review that included primary concepts, system models, multiplexing technologies, and hybrid techniques was published [16]. Many aspects, namely challenges [26], practical constraints [27], and security aspects [28] in hybrid FSO/RF literature, were also investigated. In particular, FSO/RF systems can achieve a high data rate and reliable wireless communication system.

Recent research has increasingly focused on the practical applications of hybrid FSO/RF systems. For instance, military [29], IoT [30], cellular backhaul [31], UAV hovering [32], space-air–ground [33,34,35] applications. Among recent innovations, machine-learning (ML) technology has been increasingly applied to hybrid systems. Lapčák et al. [36] proposed the use of ML techniques for predicting atmospheric channel conditions in hybrid FSO/RF systems. Additionally, Khalid et al. [37] conducted a survey on ML-supported hybrid FSO/RF systems, specifically focusing on their performance in foggy and smoggy environments. However, many existing surveys lack visual hybrid models and fail to adequately cover recent advancements in the field. To address these shortcomings, this survey aims to provide graphical representations and incorporate the latest developments in hybrid FSO/RF systems.

### 1.1. Contribution

This paper explores the published literature in the “Hybrid FSO/RF” area, covering different models and experimental tests. It provides an overview of recent developments in the hybrid FSO/RF technology, including mathematical models and real-world implementations. It also categorizes FSO/RF switching mechanisms, namely hard switching, soft switching, and relay-based mechanisms.

To support conceptual clarity, the paper also presents simplified visual models that represent the core ideas from existing literature.

Furthermore, various hybrid system architectures are discussed in diverse operational contexts, including terrestrial, non-terrestrial, and space-based communication networks, as well as machine-learning-integrated frameworks.

Finally, the paper outlines critical challenges in the field and proposes possible future research directions to guide ongoing improvements in hybrid communication systems.

### 1.2. Paper Organization

The remainder of the paper is structured as follows: Section 2 introduces related works and fundamental concepts of RF, FSO, and hybrid technologies. Section 3 discusses the evaluation metrics for hybrid FSO/RF links. Section 4 presents the classification of hybrid systems, including hard switching, soft switching, and relay models. Media access modes, machine-learning applications, and application scenarios are covered in Section 5, Section 6, and Section 7, respectively. Practical implementations are examined in Section 8, while open challenges and future research directions are discussed in Section 9. Lastly, the survey conclusion is represented in Section 10.

## 2. Related Works

This section provides a comprehensive review of recent literature on the key concepts related to RF, FSO, and hybrid FSO/RF systems. The characteristics and limitations of both RF and FSO technologies, as summarized in Table 1, are discussed. Furthermore, the dynamic FSO/RF switching mechanism and its performance metrics are examined. While specific components of the hybrid system—such as antenna design, security protocols, light sources, photodetectors (PD) [16], modulation techniques [38], frequency allocation [31], and safety considerations [39]—fall outside the primary focus of this study, a brief overview is included to maintain contextual relevance to these areas.

### 2.1. Radiofrequency (RF) Communication Characteristics

RF communication, including Wi-Fi, 5G, Bluetooth, and television broadcasts, is the most used form of wireless communication daily. It presents numerous advantages, namely the ability to penetrate obstructions, cost-effectiveness, broad coverage, and mature network architecture. Nevertheless, RF limitations, such as interference, both inter-channel and co-channel, restricted bandwidth, vulnerability to eavesdroppers and heavy rain, and spectrum regulation, should be considered.

To determine the RF performance under various conditions, mathematical models are developed to assess the system behavior. The model usually be formed in probability density function (PDF). The PDF provides the irradiance fluctuation in a turbulent channel, such as SNR, as summarized in Table 2. It is commonly applied to turbulence models such as Rician and gamma–gamma models.

The mathematical models are important in evaluating the system performance, as they provide insights into the system behavior in various environments. The following list briefly summarizes a range of fading models, each tailored to represent specific communication scenarios:

- Nakagami-m model (NM): Highly flexible for terrestrial systems, this model can adjust to different scattering and multipath fading conditions by tuning the *m* parameter. It is particularly effective in environments with diverse fading intensities, such as urban areas and satellite communications [24,40,41,42];
- Rician model: Ideal for LoS systems where a strong direct path exists. The *K*-factor is used to measure the ratio of direct to scattered signal paths. It is commonly used in satellite communications (SATCOM) and clear line-of-sight urban areas [24,43];
- Rayleigh model: Specifically designed for non-line-of-sight (NLoS) environments, this model assumes the absence of a dominant signal path. It is widely applied in densely built urban areas where buildings and obstacles cause significant scattering of signals [44,45];
- K-μ model: Often employed in complex terrain, urban areas, and autonomous mobility, this model accounts for fading caused by both multipath propagation and shadowing. The μ parameter represents the *LoS* component, while the *K* parameter quantifies the fading distribution, providing a detailed representation of varying conditions [46,47];
- α-μ model: A generalized model that adjusts the α and μ parameters to account for different fading behaviors. This model is highly adaptable, encompassing both Nakagami-m and Rician models, and is suitable for scenarios requiring flexibility in modeling fading effects [48];
- Weibull model: A less complex model used to describe small-scale fading, particularly in millimeter-wave (mmWave) communications involving significant atmospheric scattering. It is useful for various fading conditions, making it versatile for different environments [44];

These mathematical models enable the prediction of RF behavior under specific conditions, allowing for thorough analysis and evaluation prior to real-world implementation, thus supporting optimized system design and functionality.

**Table 2:** Mathematical Models for RF and FSO Systems.

System	Model	Equation	Description and Parameters
RF	Nakagami-m [24]	$f\left(x\right)=\frac{2m^{m}}{\Gamma \left(m\right)\Omega^{m}}x^{2m-1}e^{-\frac{mx^{2}}{\Omega }}$	*x*: amplitude, *m*: shape factor, Ω: mean-squared signal amplitude.
	Rician [24]	$f\left(x\right)=\frac{2x(K+1)}{\Omega }e^{-K-\frac{K+1}{x^{2}}}I_{0}\left(\frac{2\sqrt{K^{2}x+Kx}}{\Omega }\right)$	*K*: Rician factor, $I_{0}$: Modified Bessel function of the first kind.
	Rayleigh [45]	$f\left(x\right)=\frac{x}{\Omega }e^{-\frac{x^{2}}{2\Omega }}$	Ω: variance of signal amplitude.
	K-μ fading [49]	$f\left(x\right)=\frac{\mu {\left(1+\kappa \right)}^{\frac{\mu +1}{2}}}{\bar{x}\kappa^{\frac{\mu -1}{2}}e^{\mu \kappa }}{\left(\frac{x}{\bar{x}}\right)}^{\frac{\mu -1}{2}}$ $\times e^{-\frac{\mu (1+\kappa )x}{\bar{x}}}I_{\mu -1}\left(2\mu \sqrt{\frac{\kappa (1+\kappa )x}{\bar{x}}}\right)$	*K*: LoS component, μ: clustering parameter, $I_{0}$: Modified Bessel function of the first kind.
	α-μ fading [48]	$f\left(x\right)=\frac{\alpha \mu^{\mu }}{\Gamma (\mu )}x^{\alpha \mu -1}e^{-\mu x^{\alpha }}$	α: non-linearity, μ: multipath clustering.
	Weibull [44]	$f\left(x\right)=\frac{k}{\lambda }{\left(\frac{x}{\lambda }\right)}^{k-1}e^{-{\left(\frac{x}{\lambda }\right)}^{k}}$	*k*: shape parameter, λ: scale parameter.
FSO	Gamma–Gamma [50]	$f\left(x\right)=\frac{2{\left(\alpha \beta \right)}^{(\alpha +\beta )/2}}{\Gamma (\alpha )\Gamma (\beta )}x^{\frac{\alpha +\beta }{2}-1}e^{-\frac{\alpha \beta }{x}}$	α,β: turbulence parameters.
	Log-Normal [51]	$f\left(x\right)=\frac{1}{2\sqrt{2\pi }\sigma_{x}}e^{-\frac{{\left(\ln x-\mu_{x}\right)}^{2}}{8\sigma_{x}^{2}}}$	$\sigma_{x}$: standard deviation.
	Malaga [22]	$f\left(x\right)=A\sum_{k=1}^{\beta }ax^{\frac{\alpha +k}{2}-1}K_{0}\left(2\sqrt{\frac{\alpha \beta x}{\gamma \beta +\Omega }}\right)$	$K_{0}$: Bessel function parameter (α−k).
	F-distribution [52]	$f\left(x\right)=\frac{m^{m}n^{n}x^{m-1}}{\Gamma (m)\Gamma (n)}{\left(1+mx/n\right)}^{-m-n}$	m,n: turbulence parameters.
	Exponentiated Weibull [53]	$f\left(x\right)=\frac{\alpha \beta }{\eta }{\left(\frac{x}{\eta }\right)}^{\beta -1}e^{-{\left(\frac{x}{\eta }\right)}^{\beta }}$ $\times {\left[1-e^{-{\left(\frac{x}{\eta }\right)}^{\beta }}\right]}^{\alpha -1}$	x≥0 and α,β,η are the shape parameters (α,β,η>0)
	K-distribution [54]	$f\left(x\right)=\frac{2a^{\nu }}{\Gamma (\nu )}x^{2\nu -1}e^{-ax}K_{\nu }\left(2ax\right)$	ν: scatter count, $K_{\nu }$: Bessel function.
	Kim and Kruse [55]	$A=\frac{3.91}{V}{\left(\frac{\lambda }{550}\right)}^{-\delta }$	*A*: attenuation, *V*: visibility distance, delta: a constant depending on visibility condition.
	Beer-Lambert Law [56]	$A=e^{-\sigma d}$	σ: absorption coefficient, *d*: path length.

This table summarizes the mathematical models for RF and FSO systems.

### 2.2. Free-Space Optical (FSO) Communication Characteristics

FSO communication represents an advanced technology that facilitates data transmission via laser beams through free space. Compared to their high directivity and shorter wavelengths, FSO links offer high security and bandwidth. However, these advantages necessitate precise LoS alignment and increase vulnerability to atmospheric scattering and absorption. As a result, FSO systems are more sensitive to adverse weather conditions than RF systems. To tackle the LoS, scattering, alignment, and tracking challenges, Acquisition, Tracking, and Pointing (ATP, some called PAT) systems are developed. The ATP system actively adjusts the laser beam to mitigate pointing error (PE) loss.

As FSO beams propagate through unguided media, such as air and space, various factors impact the transmission quality. Therefore, to understand these effects, mathematical models are developed as follows:

- Gamma–Gamma (GG) model: Widely used to characterize the impact of turbulence on FSO link strength under strong conditions [19,46,50]. This illustrates the relation between refractive-index temperature across varying weather conditions;
- Log-normal model: Primarily applied to weak turbulence conditions, where small atmospheric particles, such as drizzle, impact FSO transmission [17,31,37];
- Malaga model: Well-suited for weak, moderate, and strong turbulence conditions, as it incorporates a wide range of variables [22,45,50];
- Snedecor model (F-distribution): Typically applied in scenarios involving strong turbulence or severe atmospheric disturbances [52,57];
- Exponentiated Weibull model (EW): Represents the limiting distribution of light intensity under weak and moderate turbulence conditions with various aperture sizes [53];
- Mie scattering model: Characterizes light scattering to predict signal attenuation resulting from adverse weather conditions, such as haze and rain [31];
- Kim and Kruse models: Widely used to predict fog attenuation based on visibility distance [37,55];
- K-distribution model: Widely applied to strong atmospheric turbulence [22,58];
- Beer-Lambert law: A fundamental principle used to estimate signal attenuation due to atmospheric absorption and scattering effects, including those caused by clouds and dust [56].

All these mathematical models are shown in Table 2. The table summarizes the mathematical models, showing each equation and the corresponding variable definitions.

**Figure 1:**
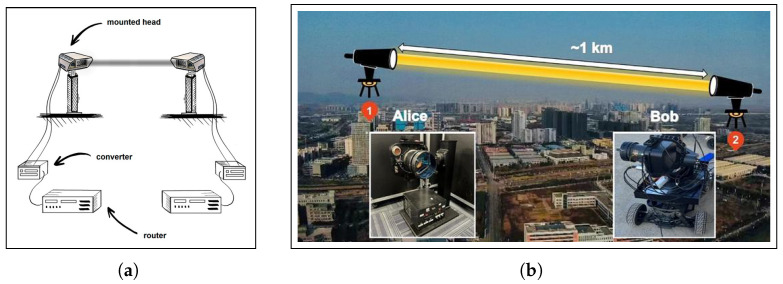
A FSO concept and prototype implementation. (**a**) an FSO diagram [66]; (**b**) Real-world prototype setup of an FSO link [67].

To validate mathematical models, experimental evaluation of FSO systems is essential. FSO communication, which uses a narrow optical beam to transmit data through free space, has been proved [59,60,61,62,63,64,65]. Figure 1 shows the basic setup, where an optical source with a lens transmits light through the atmosphere.

However, other factors, such as zenith angle, wind speed, and beam wandering, significantly influence FSO performance. Also, many mathematical models are suited to specific environments, such as the EW for varying aperture sizes and the GG for strong turbulence. No single model captures all influencing factors. Therefore, some of the equations have been further developed to cover all these factors.

### 2.3. Hybrid FSO/RF

As a standalone system, neither FSO nor RF technology is adequate for all scenarios and weather conditions. The RF technology encounters limitations in heavy rain, co-channel interference, and restricted bandwidth, while FSO is significantly impacted by atmospheric turbulence and conditions, namely fog, haze, and dust. The limitations of each technology can be addressed by integrating them into a hybrid FSO/RF system, which can leverage the strengths of both approaches.

The integration of FSO and RF systems is commonly referred to by several terms, including hybrid FSO/RF, mixed RF/FSO, FSO/RF integration, and dual-mode FSO/RF systems, all of which denote the same concept. This integration can be configured in distinct modes: hard switching (HS), soft switching (SS), relay, and multiple-access models, which will be discussed in Section 4 and Section 5, respectively.

A fundamental component of the hybrid system is a switching mechanism that dynamically alternates between FSO and RF links based on channel conditions. Decisions regarding this switching are typically based on specific threshold levels, including SNR and Received Signal Strength Indicator (RSSI). When these thresholds fall below a predetermined value, the system transitions its operational mode to ensure overall reliability and performance.

- SNR: Explains the quality of a communication link. It is the ratio of the signal power to the noise power in the form of decibels (dB);
- RSSI: Monitors the strength of the received signal and is influenced by environmental factors such as distance, obstacles, and weather conditions.

Another essential component is the data feedback mechanism, referred to as Channel State Information (CSI), which enables the system to monitor channel conditions. CSI provides insights into the properties of the channel, allowing the hybrid system to assess the environmental situation and select the appropriate operational mode.

**Figure 2:**
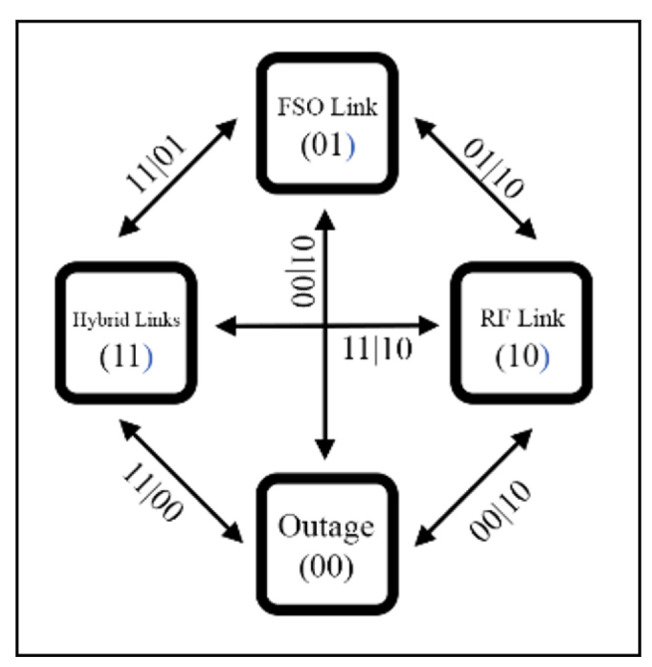
A CSI transition states: Hybrid Link (11), FSO Link (01), RF Link (10), and Outage (00).

Numerous studies are currently underway on CSI methods. For instance, Shivtarav et al. [68] investigated a one-bit feedback system to control the hard switching mechanism between FSO and RF links, where CSI is critical, as improper switching can significantly impact system performance. Their analysis indicates that enhancing the feedback SNR mitigates the effects of fading and noise. Another study [69] examines the impact of errors associated with two-bit CSI on system performance. The soft-switching scheme [56,70,71], which employs a two-bit CSI, operates a four-mode hybrid system (illustrated in Figure 2). However, environmental factors affecting CSI can lead to incorrect mode switching, thereby reducing performance. These findings emphasize the importance of reliable CSI feedback to sustain optimal system performance.

Although CSI is important, the system faces another challenge called the ping-pong effect. The effect will arise when rapid changes in channel conditions cause the system to frequently switch between FSO and RF links. This led to destabilization in the connection and system outage. To mitigate this issue, numerous researchers have proposed solutions, such as implementing time delays [65,72,73] or utilizing dual thresholds [74], to stabilize the switching process.

In summary, the hybrid FSO/RF system effectively leverages the strengths of both FSO and RF technologies by selecting the optimal link based on switching thresholds, such as SNR and RSSI. Reliable CSI modules, which provide feedback on switching conditions, play a crucial role in this selection process. Consequently, these modules are essential for maintaining system efficiency, particularly during critical events.

## 3. Performance Metrics

In evaluating system performance, several technical factors must be considered to assess overall efficiency. These factors include throughput, power consumption, link availability, operational range, Quality of Service (QoS), latency, and packet loss. Most studies assess these metrics using adapted forms of probability density function (PDF) equations. For example, the Cumulative Distribution Function (CDF), which represents the probability that a random variable x is less than or equal to a given value, is often used to determine the outage probability (OP) of a system. Additionally, Shannon’s theory can be applied to the PDF to calculate system capacity.

To simplify and generalize the complex equations, many studies transform the original PDFs using special functions such as Fox’s H-function or Meijer’s G-function, which offer greater flexibility in modeling wireless communication channels [75]. As a result, system performance metrics are derived by converting the PDF into more practical forms such as OP, average BER, or capacity—to facilitate performance evaluation, as follows:

- Outage Probability (OP): OP measures the likelihood that signal quality will fall below an acceptable threshold, thus indicating the potential for system failure;
- Throughput: This metric reflects the system’s successful transmission rate, indicating data transfer efficiency and speed;
- Bit Error Rate (BER): BER measures the ratio of incorrect bits to the total number of transmitted bits, serving as an indicator of reliability;
- Symbol Error Rate Probability (SEP): Similar to BER, SEP measures per-symbol errors, particularly relevant in high-order modulation systems;
- Ergodic Capacity (EC): EC represents the average data transfer rate per channel, providing insights into the system’s ability to allocate data across channels;
- Latency and Jitter: Latency refers to transmission delays, while jitter denotes variations in packet arrival time. Both metrics are critical for real-time applications.

By evaluating these performance indicators, one can understand the trade-offs inherent in system design. For example, extending the operational range may reduce reliability, while optimizing throughput might increase power consumption. Each design must prioritize specific goals, and researchers should select appropriate designs based on their objectives.

## 4. Classification of Hybrid Models

Switching mechanisms on hybrid FSO/RF systems can be classified into three categories: hard switching, soft switching, and relay-based configurations. Each mechanism has its own advantages and disadvantages. Consequently, engineers must carefully design and select the most appropriate switching mechanism to suit the specific requirements of their system. This section provides an overview of the Switching mechanisms’ advantages, disadvantages, associated techniques, and relevant literature to understand their design and application.

### 4.1. Hard Switching Model (HS)

**Figure 3:**
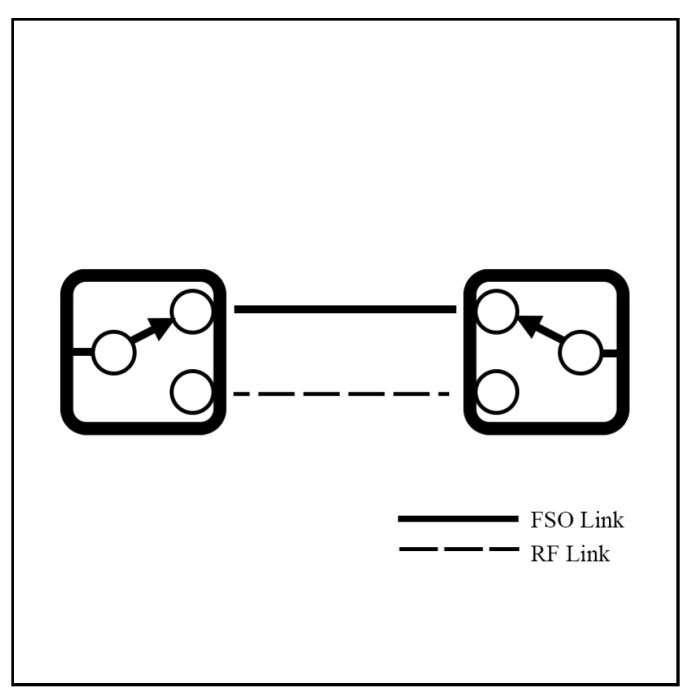
Example of a hard switching mechanism.

In a system based on HS, either an FSO or RF link is operated at a certain time, as shown in Figure 3. This figure represents how HS operates through ON/OFF switching between FSO and RF links. The rapid switching between FSO and RF can trigger the ping-pong effect. This effect introduces instability, latency, and packet loss in the system. To address the effect, CSI is used to monitor channel conditions between the two links to enable more effective switching and prevent the effect. Despite these challenges, HS is simple to implement and cost-effective, making it an attractive option for simple applications that do not require complex algorithms.

Several researchers have investigated the HS technique. Kirubakaran and Selvaraj [46] proposed an HS hybrid FSO/RF system based on GG/κ-μ distribution models. The authors also examine the impact of RF channel interference on the hybrid system. Their findings reveal that RF interference adversely affects both OP and SEP performance. To mitigate this issue, they propose the use of an optimal SNR switching threshold as a potential solution.

Vishwakarma and Swaminathan [76] proposed an HS hybrid FSO/RF system under the Malaga/α-η-κ-μ distribution models with various modulation schemes and PE impairment. Their study highlighted the α-η-κ-μ model, which combines NM, α-μ, and κ-μ models’ ability to capture a range of fading behaviors and predict performance accurately. Their simulations indicate that the backup RF link can enhance SEP performance even under severe PE, attenuation, and turbulence.

**Figure 4:**
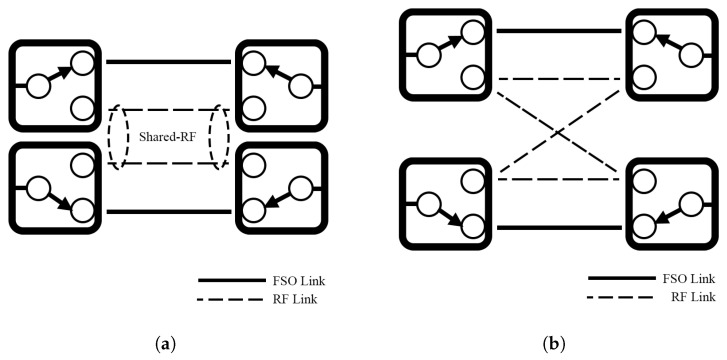
System models: (**a**) Shared-RF system model, (**b**) On-Demand hybrid system.

Nath et al. [40] establish novel architectures of shared RF (Figure 4a) and on-demand RF hybrid (Figure 4b) solutions for HS hybrid FSO/RF systems. The shared-RF model uses a single RF channel that is continuously available as a backup for multiple FSO systems. In contrast, the on-demand RF hybrid model only activates the RF channel when needed. Once the FSO system no longer requires the RF backup, the channel is released and becomes available for use by other FSO systems. The shared-RF system with cognitive radio is evaluated in [77]. The system shares an RF backup with a cognitive mode between two FSO links. The RF sharing mechanism uses energy sensing and interweaved cognitive mode to detect FSO links. If an FSO link fails, the system activates the RF backup link to allocate traffic. This sharing can reduce the OP and RF license costs while increasing the utilization performance of the RF resources.

Furthermore, an improvement of the cognitive hybrid system is proposed in [78]. The system aims to prevent RF interference by scanning the licensed RF spectrum and identifying usable frequencies. The simulation results indicate that the cognitive radio with a shared RF improves EC, OP, and BER performance.

Another novel architecture, the on-demand solution, is presented in [79]. The system comprises two individual FSO links with two backup shared-RF links. However, the on-demand system’s advantage is very small despite mutual RF resource sharing, as shown in their simulation results. Lastly, the interference effect on their RF cognitive system is examined in [80]. The results show that severe interference can negatively affect BER and OP performance. However, an RF cognitive system can mitigate these issues. As a result, the cognitive hybrid FSO/RF system can be a valuable approach for enhancing performance.

Despite numerous studies on HS, there remains a lack of standardized protocols for switching between RF and FSO links, which can result in interruptions in continuous connectivity. While much of the existing literature focuses on system design, the comparison between theoretical models and real-world implementations remains underexplored. These gaps present opportunities for further research and experimental validation.

### 4.2. Soft-Switching Model (SS)

**Figure 5:**
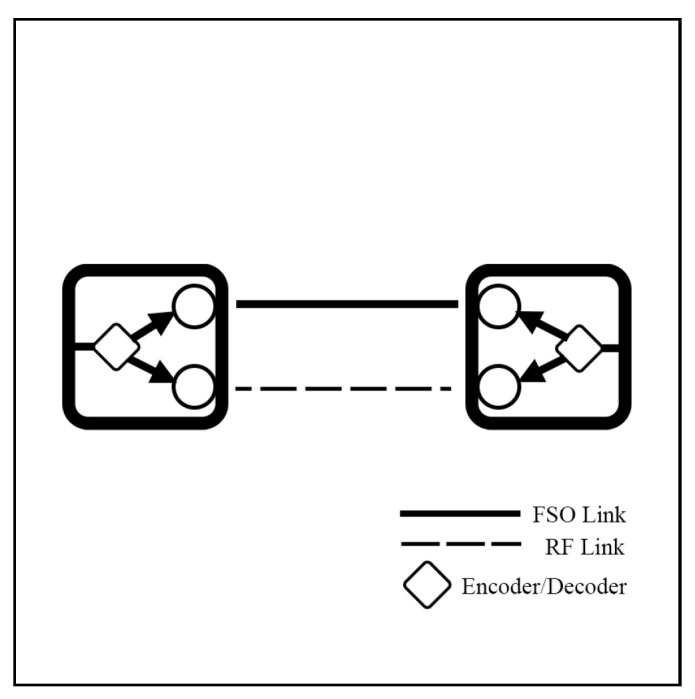
Example of a soft-switching mechanism.

A soft switching model (SS) operates on both FSO and RF links at the same time. It distributes the data across both links based on various factors such as network setup, throughput, link quality, and environmental conditions, as shown in Figure 5. Depending on the SS implementation, data streams can either be split between the two links to increase the throughput or duplicated to improve reliability.

In the SS mechanism, CSI is utilized not only to monitor link conditions but also to adjust the data transmission on each link. By facilitating simultaneous data transfer across both links, SS can decrease the likelihood of the ping-pong effect and enhance overall system performance. However, this setup is more complex setup and consumes more power than HS to ensure functionality, particularly in highly dynamic environments.

Various models exist for data transmission using SS schemes, including parallel links, adaptive modulation, load-balancing, signal selection, and coding. Each of these models contributes unique attributes to the overall system. Several studies have demonstrated the benefits and effectiveness of SS schemes.

Short-length Raptor codes were designed and implemented in the hybrid FSO/RF link [55]. The code packets are sent on both links simultaneously, achieving a 714 Mbps data rate with a 97 mW power consumption. This code can increase the reliability of the network but reduces throughput due to the header code in the system.

A soft-switching FSO/THz-RF system based on an outage probability algorithm was proposed in [48]. The algorithm checks the outage threshold values of each system and selects the appropriate link for each scenario. The results show that the system can improve reliability while increasing the complexity of system design.

Shao et al. [43] investigate an adaptive modulation scheme for soft-switching hybrid FSO/RF links using a random forest machine-learning algorithm under various environments. The FSO link has three modulation modes: M-PSK, M-QAM, and M-pulse-phase modulation (PPM), while RF has two modes: M-PSK and M-QAM. The system is trained using 2-year-weather data and BER calculations from the data. The simulation results indicate that a soft-switching strategy can enhance reliability (OP) in real time under varying weather conditions.

Shrivastava et al. [70] introduce a new switching scheme for hybrid FSO/RF using adaptive combining under negative exponential fading/Rayleigh fading channel models. There are three threshold levels for transmission modes: The primary mode is the FSO link, the second mode is a combination of FSO and RF links, and the last mode is the RF link only.

Other atmospheric models are investigated in [52,56,81,82] under Malaga/κ-μ distribution, F-distribution/NM with PE, GG/exponential fading models with PE, and GG/Rician-NM models with PE, respectively. These simulation results show that adaptive combining can effectively transmit and combine signals over both FSO and RF links without degrading RF power utilization and FSO link quality. This leads to high capacity and improved reliability (OP, BER) and EC performance.

Moreover, the optimal selection of these threshold levels via adaptive combining has been further studied in [71]. This approach aims to balance OP, SNR thresholds, and power consumption. They suggest that to achieve optimal performance, the algorithm should focus on minimizing BER and maximizing SNR while maintaining a fixed OP threshold.

In summary, SS schemes facilitate continuous data transfer by selecting or combining signals based on various criteria. This scheme can significantly reduce outage time and ping-pong effects. Numerous studies have shown that techniques such as coding, load-balancing, and adaptive modulation improve reliability but slightly reduce throughput. Although SS is designed to use both FSO and RF links, the significant difference in throughput between the two often prevents full cooperation. In most studies, the RF link serves as a shared or duplicated link, which limits the system’s overall capability. Furthermore, FSO links are typically ineffective in foggy conditions, while RF links suffer in heavy rain. These scenarios prevent the system from reaching its full potential. To the best of our knowledge, this limitation in SS mechanisms has received little attention in the existing literature.

### 4.3. Relay Switching Model

**Figure 6:**
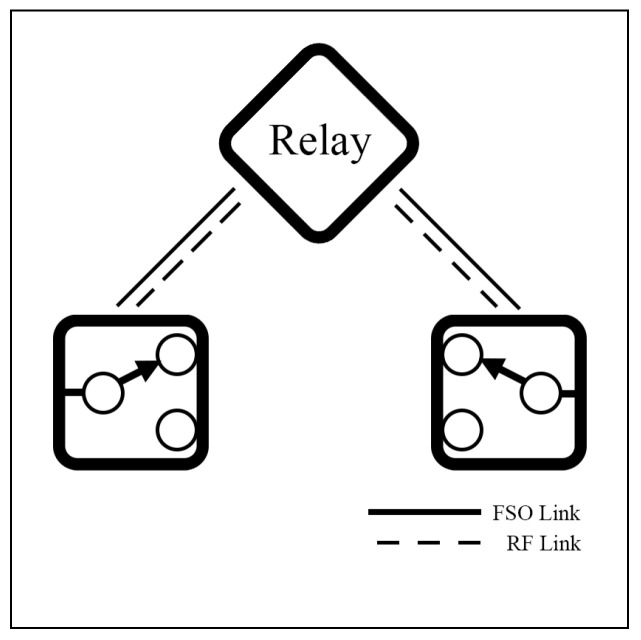
Example of a relay switching.

To improve coverage range, reliability, and robustness, a relaying technique is suggested. In a relay setting, FSO typically serves as the backbone network for high-capacity transmission, while RF operates as an access network to handle obstructed paths. Relay systems can be deployed in unreliable environments, such as satellites or UAVs in aerial networks or during adverse weather events. However, the relay system faces challenges, such as increased latency and costs. To sum up, relay-assisted hybrid FSO/RF systems offer a practical solution for extending connectivity in challenging environments. Figure 6 shows an example of a relay in a hybrid system. In this setting, the relay is used to extend coverage or redirect the communication path, while the system can dynamically select between FSO and RF links based on link conditions.

A relay mechanism can be classified into three categories, including amplify-and-forward (AF), decode-and-forward (DF), and intelligent reflecting surfaces (IRS), each offering distinct trade-offs.Numerous researchers have proposed these techniques in various publications.

Sun et al. [83] examine a relay hybrid FSO-RF system under GG/α-F-distribution fading models, accounting for PE impairment. The system utilizes a relay station to switch between FSO and RF signals. Additionally, the study compares AF and DF relaying types using intensity modulation with direct detection (IM/DD) and heterodyne (HD) detection methods. Numerical results indicate that HD and DF schemes can effectively mitigate the negative effects of fading, providing superior performance in terms of OP, BER, capacity, and EC compared to IM/DD and AF schemes. However, these approaches entail higher costs and complex designs, and performance still degrades significantly under conditions of strong atmospheric turbulence and severe PE.

Ninos et al. [41] introduced a full-duplex DF relay HS hybrid FSO/RF under GG/NM models with PE, beam wander, residual SI (RSI), and IQ imbalance (IQI) impairment to extend overall link ranges and performance. The study identified that the major limitations in OP performance are caused by atmospheric turbulence, IQI, and RSI impairment because of the possibility of backup RF connection loss. Furthermore, the study demonstrates that the appropriate selection of beam parameters is crucial for achieving optimal performance in conditions of weak to moderate turbulence.

**Figure 7:**
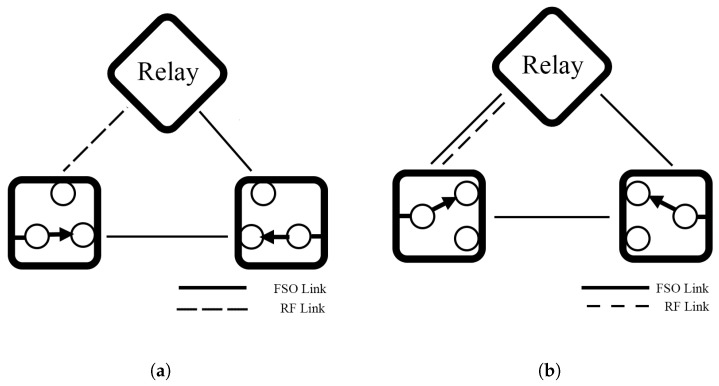
Hybrid relay systems: (**a**) RF backup link model, (**b**) FSO/RF backup link.

A novel hybrid FSO/RF-FSO system with an AF relay is designed [74]. The architecture includes two links: directly and indirectly connected to an Rx. The direct link connects a Tx to an RX via an FSO link only while the indirect connection is through the relay, which can convert between FSO and RF signal as a backup link as shown in Figure 7a. The system operates with dual SNR link-switching thresholds under GG/Rayleigh [74], Malaga/Rayleigh [45], and F-distribution/NM [57] models. The dual threshold separates the switching mechanism into three modes: a primary link, a backup link, and an outage mode. These mathematical equation results show that the dual threshold can prevent unnecessary switching called the ping-pong effect and the system can improve OP, BER, and EC performances compared to the FSO-only system in all weather conditions.

Additionally, Bag et al. [84] present an improved system by changing an AF relay system [45] to a DF relay and adding another FSO connection in the first hop of the backup link as illustrated in Figure 7b. The system is assessed under Malaga/NM models, and different weather conditions affect each link differently. The numerical results show that the new backup link can significantly enhance system EC and improve OP and BER performance when the primary link faces severe weather conditions. Also, a DF can eliminate noise and non-negativity problems but trades with more complexity.

**Figure 8:**
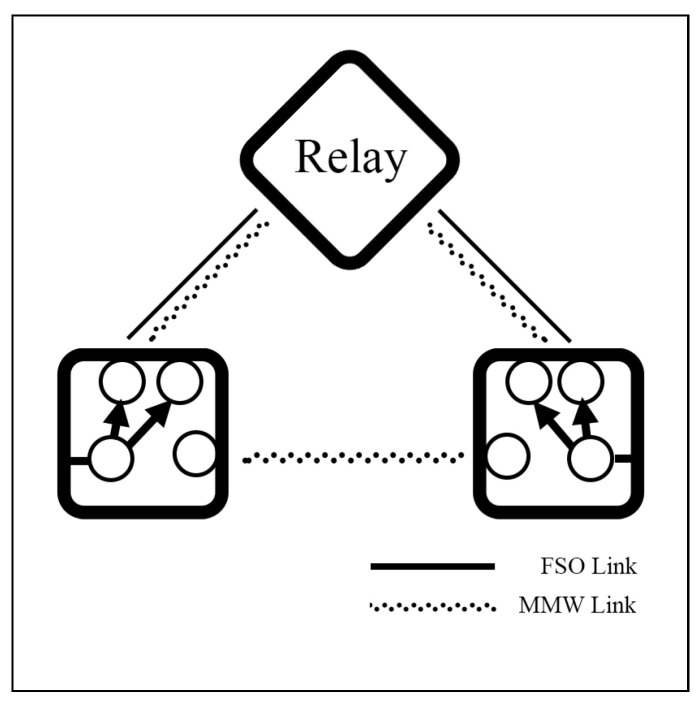
Double threshold hybrid FSO-mmW system model.

Mogadala et al. [85] propose a relay system that employs switching between FSO and mmW links under the log-normal/NM models. In this system, the main link integrates FSO and mmW links connected through a relay using a selection combining (SC) scheme, while the backup link connects to Rx via the mmW link directly, as depicted in Figure 8. The numerical results demonstrate that the system provides better OP performance than a conventional FSO-RF relay system in [74].

IRS is a passive relay solution that can improve system coverage. It consists of many reflecting elements (often called meta-surfaces) that control the signal propagation. However, it does not amplify the signals and requires precise control to reflect them. Numerous studies have explored the potential of the IRS in hybrid systems.

**Figure 9:**
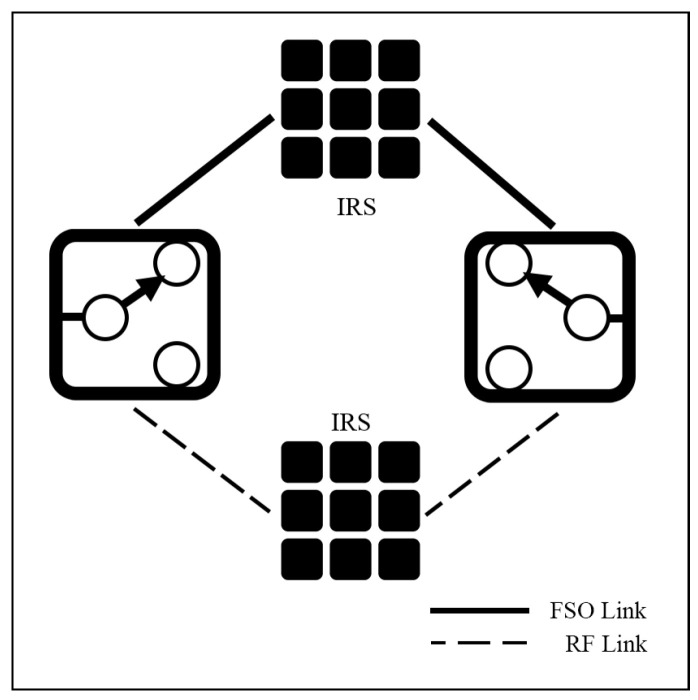
IRS-assisted hybrid FSO/RF system.

In [42,86], an IRS-assisted hybrid FSO/RF system is studied. The system is simulated under GG/Rayleigh [86] and GG/NM models with SC scheme [42]. Both FSO and RF systems add the IRS to their relay subsystems as represented in Figure 9. From their numerical simulation results, it is observed that IRS can be used as efficient relays for both links, and increasing the number of reflecting elements can improve OP, BER, EC, and SEP performance significantly.

Verma et al. [87] developed an IRS-assisted mixed DF relay FSO-RF system with a hybrid automatic repeat request (H-ARQ) protocol. The system is simulated under GG-Rician models to estimate OP and PER performance. The study finds that increasing the H-ARQ transmission round can enhance performance. This design highlights the potential of IRS and H-ARQ to mitigate system issues even when struggling with high PE, NLoS, and strong turbulence.

**Figure 10:**
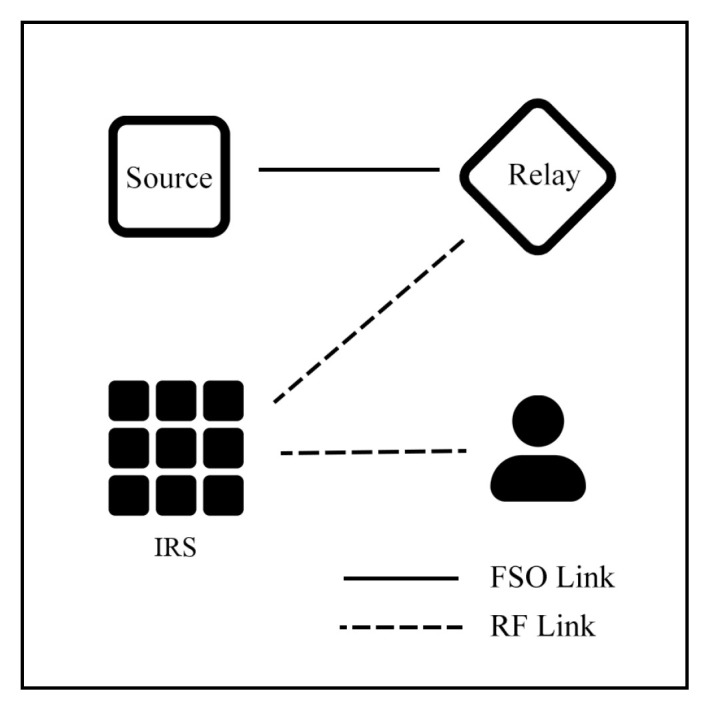
IRS with relay system.

Sun et al. [88] analyzed the end-to-end performance of hybrid FSO-RF-IRS with imperfect CSI as shown in Figure 10. They also compared AF and DF relay types and HD and IM/DD schemes. The equations were derived to analyze OP, BER, and EC performance. The findings indicate that the performance primarily relies on IRS performance with a minor effect on the FSO parameters of the proposed system.

**Figure 11:**
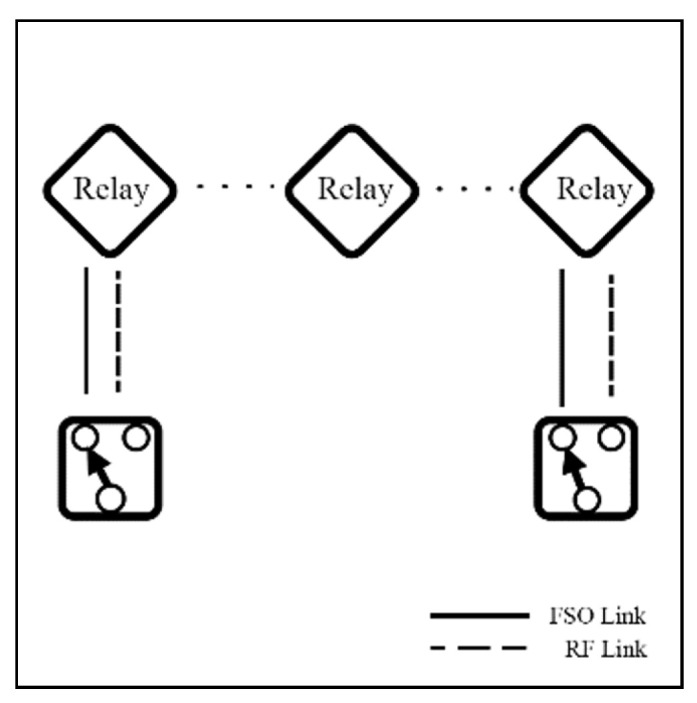
Multi-Hop FSO/RF system model.

Altubaishi and Alhamawi [89] propose an AF Multi-Hop FSO/RF system under GG/NM models with PE effect, as shown in Figure 11. Building on this, the authors [90] replaced AF with DF relays in the Multiple-Hop system. Both systems [89,90] present an adaptive number of relay hops (Multi-hops) to maintain a stable connection from Tx to Rx, mitigating the impact of severe turbulence. The results indicate that, by increasing the number of relays, the capacity performance rises as well because of the enhancement of the average SNR per hop. Since multiple relays can reduce atmospheric effects, FSO links rarely degrade from the conditions. Therefore, the RF backup links become unnecessary if the FSO link consistently maintains good quality. This implies that RF links can be redundant because of the multiple relay technique.

A relay can improve the coverage, reliability, and performance of a hybrid FSO/RF system. AF relays are simple, amplifying the received signal, while noise also amplifies as well. In contrast, the DF technique reduces noise before forwarding the received signal, though it is more complex. IRS provides a passive solution by controlling signal propagation without amplifying the signal. Each relay type has unique properties, but factors such as latency and jitter should be considered when integrating these relays into the system. However, all the aforementioned studies lack comparison with real-world scenarios. For example, IRS is often regarded as innovative relay technology, yet rarely implements it. Additionally, jitter and latency metrics are evaluated scarcely despite their significant impact on overall system performance. This may be due to the absence of well-defined mathematical models for assessing these metrics.

In conclusion, This section classifies hybrid FSO/RF systems into three models. HS is simple but vulnerable to performance issues like the ping-pong effect. SS provides continuous data transfer by simultaneously operating both FSO and RF links, though it increases complexity and power requirements. Moreover, the relay mechanism can increase coverage and reliability but increases latency and costs, too. Lastly, the literature summary of this section is presented in Table 3. The table shows the research on hybrid FSO/RF systems over the last 5 years, which focuses on switching and relay mechanisms, categorizing studies into hard switching, soft switching, and relay mechanisms. Also, it shows their objectives, simulation models, evaluation metrics, and contributions.

## 5. Multiple-Access Models

This section explores various multiple-access models and techniques in hybrid FSO/RF systems, including Single Input Multiple Output (SIMO), Multiple Input Single Output (MISO), and Multiple Input Multiple Output (MIMO). Multiple-access models in which multiple users share communication mediums are necessary for high-density environments. Many techniques, such as Non-Orthogonal Multiple Access (NOMA), Spatial Modulation (SM), and transmit source selection (TSS), are deployed. Each model focuses on handling multiple users/apertures, optimizing signal transmission, and improving overall system performance.

**Figure 12:**
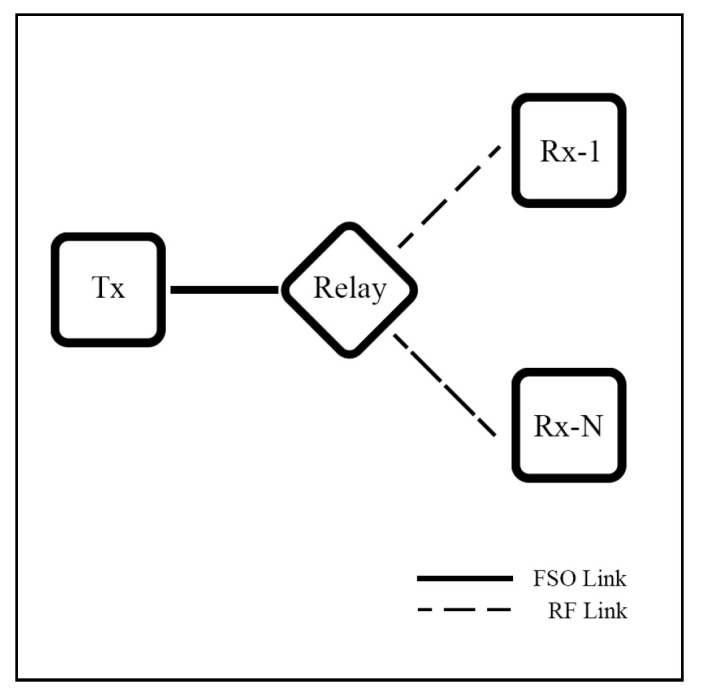
SIMO hybrid FSO/RF system model.

Nguyen and Le [92] investigate a SIMO (1×N apertures) hybrid FSO/RF with DF relay with NOMA. The system is simulated under GG/Rayleigh and Malaga/NM models to identify user fairness. The relay converts the signal between FSO and RF, where the first hop is the FSO link, and the second hops are the RF links with NOMA techniques, as illustrated in Figure 12. Building on this system, Nguyen et al. [93] further explore the SIMO hybrid-NOMA system with an AF relay using a perfect successive interference cancellation (SIC) technique. Using SIC can mitigate interference between users to improve performance and ensure the fairness of two users. Also, authors [94] study this model with a power allocation factor. Consequently, the FSO/RF-NOMA system can improve EC and support multiple users in high-density environments, while performance is influenced by power allocation, weather conditions, and SNR thresholds.

**Figure 13:**
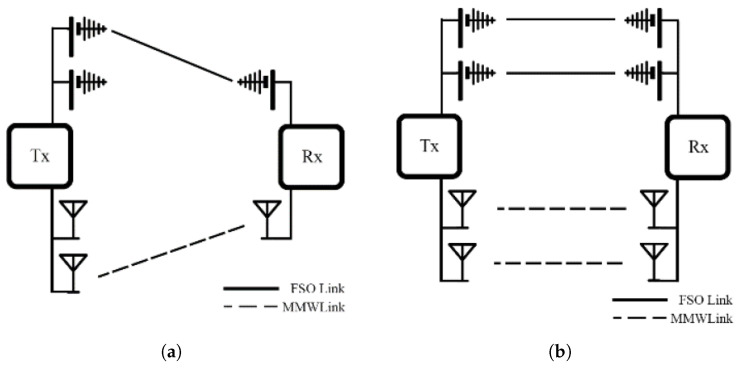
Multiple aperture hybrid models: (**a**) SIMO system, (**b**) MIMO system.

Sharma et al. [95,96,97] introduce multiple aperture hybrid FSO/mmW with a transmit aperture selection (TAS) scheme based on the adaptive modulation scheme. In [95], a MISO (N × 1 apertures) hybrid system under GG/κ-μ fading under M-PSK modulation is presented. The system consists of multiple FSO/RF apertures at the Tx station and an FSO/RF aperture at Rx, as shown in Figure 13a.

Moreover, to enhance the MISO system, the concept of space shift keying (SSK) is introduced [96]. Under the Malaga/κ-μ fading models simulation using multi-pulse-amplitude modulation (M-PAM), the MISO hybrid system using the SSK technique outperforms the individual FSO system in terms of OP, SEP, and BER performance. Additionally, the MISO model can improve reliable high-data-rate communication and aperture misalignment robustness.

Furthermore, in [97], the MIMO (N × N apertures) technique with the TAS/SC scheme is introduced, as shown in Figure 13b. The multiple antennas for FSO and RF are deployed, and diversity gain under the Malaga/κ-μ fading is analyzed. This shows that the TAS algorithm selects the best-receiving signal from each aperture and chooses the aperture based on the SC technique. The mathematical results reveal that this system can achieve more reliability and higher capacity compared to the individual MIMO system.

Mog and Kshetrimayum [98] present a MIMO hybrid FSO/RF system with the optical space shift keying modulation (OSSK) technique. The OSSK can enhance spectral efficiency by selectively activating one of several laser diodes to transmit data. Consequently, the system achieves a higher OP, EC, and SEP performance than systems using only FSO or RF communication under Malaga/NM models.

Lv et al. [99] investigate a protograph of low-density parity-check (PLDPC) codes for the MIMO hybrid system. This approach minimizes errors across FSO and RF links under changing weather conditions. The PLDPC detects and corrects errors in data transmissions, while the adaptive interleaver dynamically rearranges the data bits before transmission, spreading out potential errors and making them easier to detect and correct. The approach can offer high reliability even under severe weather conditions.

Singh and Tiwari [72] inspect a Time-Hysteresis (TH) switching-based cooperative 2×2 MIMO DF relaying hybrid FSO/RF system with a majority logic combining (MLC) algorithm. The TH switching manages link selection by increment delay time to prevent the ping-pong effect. Also, the MLC framework compares the received data from each MIMO FSO and RF subsystem to select the most reliable data. Numerical results indicate that the MIMO with TH and MLC approach can strengthen overall performance and achieve SEP $10^{-6}$ in the lower SNR region.

**Figure 14:**
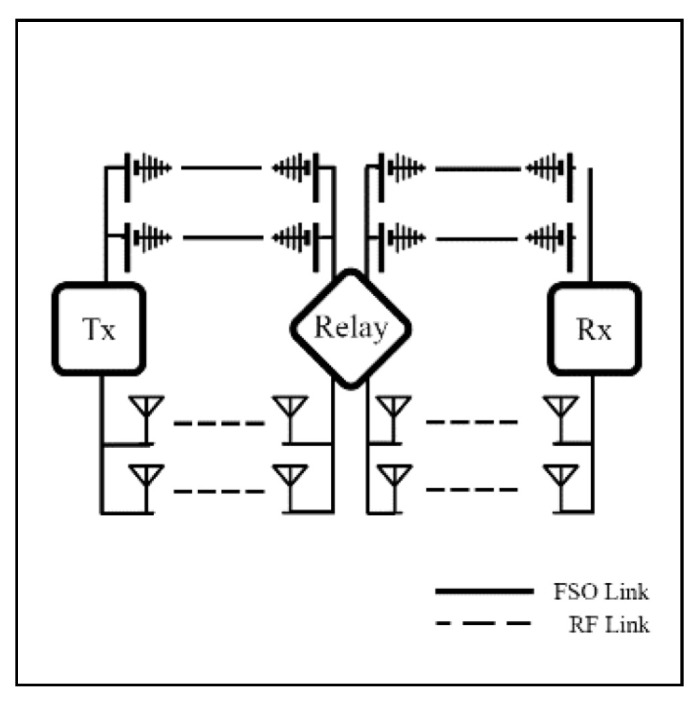
MIMO relaying hybrid FSO/RF model.

Bhowal and Kshetrimayum [100] propose a relay-based hybrid MIMO FSO/RF that integrates hybrid spatial modulation (HSM) and transmit source selection (TSS), as shown in Figure 14. The HSM can reduce signal interference using a single source based on the automatic message control bit without considering channel conditions. To resolve this limitation, TSS is introduced, where the source is selected based on the channel conditions to optimize the performance. Therefore, combining HSM with TSS can optimize both FSO and RF links by selecting the best sources. Simulation results show that the HSM with TSS system is the most effective method for improving OP performance in multiple antenna systems by comparing it with a single HSM or TSS system.

Yang and Liu [101] investigated a hybrid FSO/RF using HF and SC techniques to improve BER and OP performance. The F-distribution and NM channel were employed for FSO and RF, respectively. The study shows a comparison between modulation types, including 16PAM, OOK, BPSK, and 16PPM. The results indicated that the 16PPM technique is better than the other modulation schemes.

Shakir [102] shows a novel hybrid FSO/mmW using an SC scheme without requiring CSI under GG/NM models. The system simultaneously transmits data over both FSO and RF links at the same data rate (parallel links), which are then combined at the Rx. This model is also tested under a foggy weather channel [103]. The approach maximizes the advantages of both links while avoiding the drawbacks of the ping-pong effect. The performance results of the proposed system [102,103] efficiently enhance OP and BER performance across various turbulence conditions.

Wu et al. [104] evaluate a hybrid FSO/mmW using an MRC scheme under GG/Rician fading models. In this system, the data are transmitted concurrently over both FSO and mmW links and are combined at Rx via MRC. Their numerical results show that the hybrid FSO/RF system with an MRC scheme outperforms a single FSO link or RF-only link in terms of OP and BER performance.

Huang et al. [47] studied a comparison between the MRC and SC schemes in SS hybrid FSO/RF systems under GG/κ-μ fading models. Their study evaluates OP and BER performance across various configurations, including adaptive quadrature amplitude modulation structure (M-QAM), detection type (IM/DD or HD), RF antenna selection (SISO or MIMO), and diversity combining (MRC, SC). The numerical results indicate that the hybrid FSO/RF system using MIMO RF, HD, and MRC has better OP performance compared to other combinations in an ideal condition, although with increased system complexity.

Roumelas et al. [44] investigated a triple hybrid FSO/RF/mmW system with an SC scheme under negative exponential distribution/Rayleigh/Weibull models. The study evaluates OP performance with consideration of the PE effect. Their simulation results proved the potential of the triple hybrid system while showing it outperformed the traditional FSO/RF hybrid system.

To sum up, multiple-access models, such as SIMO, MISO, and MIMO, enable the handling of data streams and multiple users. Each model brings trade-offs between complexity, costs, and performance that are suitable for different operational scenarios. Ongoing research is dedicated to refining system efficiency, reliability, and resilience against environmental challenges.

Diversity techniques, including SC, MRC, and EGC, are evaluated in hybrid FSO/RF. The SC technique identifies the signal with the highest SNR, while the MRC integrates multiple signals with weights proportional to their respective SNRs. The EGC technique, however, combines signals with equal weights following phase alignment. These techniques enhance the reliability, capacity, and quality of hybrid FSO/RF systems in dynamic environments.

Again, most existing literature relies heavily on mathematical modeling, with limited comparison to real-world implementations. In addition, many protocols have been developed for specific purposes. For example, NOMA for RF systems [92], TAS for aperture selection [95,96,97], and TSS for selecting the best transmission source. However, there is a lack of integration among these protocols and a limited comparative evaluation across different schemes. Furthermore, comprehensive studies examining the performance of various diversity techniques under different environmental scenarios remain unexplored.

## 6. Machine-Learning Applications

In recent years, ML has gained significant research interest. Many applications adapt ML to enhance performance. The hybrid FSO/RF also integrates ML models, such as decision trees, reinforcement learning, and deep learning algorithms, demonstrating high prediction accuracy for weather conditions. These advancements significantly improve reliability and adaptability to varying climates.

Haluška et al. [61,105] conducted an experiment to predict weather conditions affecting an HS hybrid FSO/RF using a decision tree ML algorithm. The experiment was tested under a 230-m range. The weather data collected from the RSSI sensors included temperature, humidity, airborne particulate matter, and visibility. These data were processed through the decision tree algorithm, which identifies the best prediction models based on suitable training data. The model can achieve a mean square error (MSE) of 0.94, indicating a high level of accuracy in weather prediction [105].

Liscinska et al. [62] applied deep data analysis methods for hard switching in hybrid FSO/RF systems using decision tree algorithms to predict RSSI. The study employed Python-based data processing and utilized weather station data from the Technical University in Kosice. The results demonstrated that the prediction achieved 79% accuracy with a 1-min forecasting window using a 90:10 ratio of training and testing data.

Meng et al. [63] propose another predictive mechanism using the long short-term memory (LSTM) algorithm for HS hybrid FSO/RF. The model aims to minimize energy waste by proactively adjusting the link-switching mechanism. The predictive link algorithm periodically samples FSO signal power in the input dataset to reduce prediction errors. The FSO link remains in a low-power state for most of the RF transmission phase to conserve energy. Simulation results verify that the mechanism can improve energy efficiency.

Lapčák et al. [64] propose the decision tree regression method with the AdaBoost regressor to predict RSSI for HS hybrid FSO/RF. The dataset includes visibility, temperature, dense fog, wind speed, humidity, and air pressure, which are used to create an RSSI prediction model. An analysis in [62] tests different input datasets and ratios, revealing that specific weather parameters (e.g., temperature, visibility, and dense fog) significantly influence RSSI predictions. The model achieves an MSE of 0.66, and a score of 0.89 [62], successfully predicting one to two minutes ahead.

**Figure 15:**
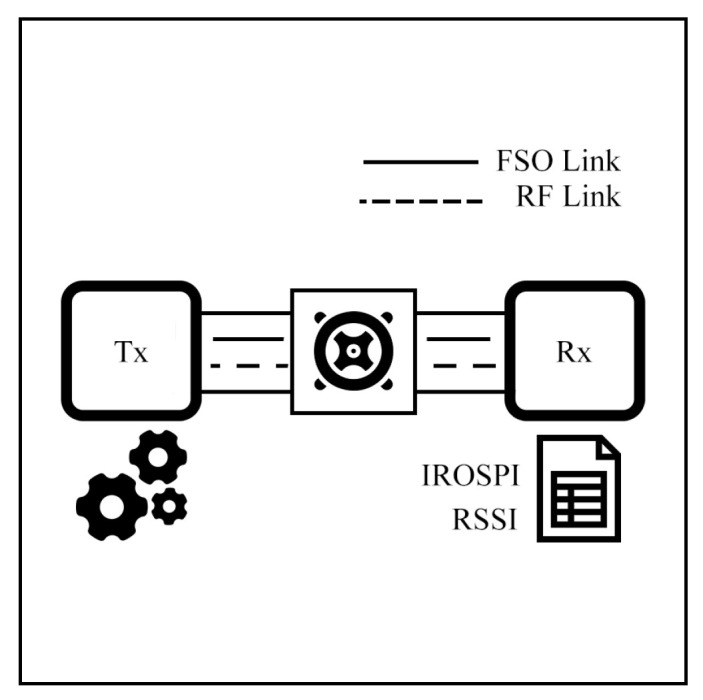
Testbed diagram of the intelligent hybrid FSO/RF experiment.

Song et al. [65] experimentally propose an HS hybrid FSO/RF system with adaptive link-switching and selection. The testbed consists of FSO, RF, optical signal power intensity (IROSPI) modules, and a chamber tube, as shown in Figure 15. The switching mechanism selects either FSO or RF based on the power received by the IROSPI. If the received power drops below a predefined threshold, the RF module is activated, and a delay timer is triggered to avoid the ping-pong effect. The experiment is implemented in a controlled chamber perfectly.

Furthermore, an upgraded system involving Recurrent Neural Networks (RNN) for RSSI prediction is presented in [106]. The primary objective is to enhance transmission effectiveness by forecasting RSSI and controlling the power strategy. This system comprises FSO, RF, a chamber tube, an RSSI detector, and power control components. The transmitting power is controlled by the Gated Recurrent Unit (GRU) technique, a machine-learning method suitable for time-series prediction. The GRU model is trained on three days and three months of weather datasets in [106,107], respectively. As a result, the RSSI change trend is predicted, allowing the effective adjustment of transmission power.

An intelligent RSSI prediction system for hybrid FSO/RF is further enhanced in [108] by incorporating a Time Attention Mechanism (TAM) into the GRU model. The TAM allows the model to assign different weights to each RSSI component of the input (e.g., temperature, humidity, partial pressure, and wind speed) in a time series. Additionally, an adaptive modulation strategy (M-QAM) is introduced to improve system reliability. The system is trained on ten years of weather data from five cities and achieves a 90% prediction accuracy for FSO channel fading, with an absolute percent error value below 3% and BER values around $10^{-3}$.

These experimental findings [65,106,107,108] confirm the successful implementation of adaptive link selection and switching mechanisms using ML models. As a result, the hybrid system can increase transmission stability and improve its ability to handle diverse environmental changes.

Machine-learning (ML) models are still underexplored in hybrid systems. While many authors have adopted ML models such as Decision Tree [62,105], AdaBoost Regression [108], and RNN to analyze weather data [64] and predict RSSI [108]. LSTM networks have also been applied to minimize energy consumption in hybrid systems [63]. However, other ML models still need to be investigated, including Vector Autoregressive with Exogenous Inputs (VARX) [109], Federated learning (FL) [110]. In addition, the types of input data used for prediction, such as meteorological (MET) data, atmospheric turbulence parameters, and FSO/RF channel conditions, require further study. Lastly, predictive modeling for parameters, including modulation order and system threshold, also needs to be explored.

## 7. Application Scenarios

From pure mathematical models to real-world simulation applications, this section explores how these models adapt to diverse scenarios through the integration of multiple communication layers. This framework can be divided into three categories, including terrestrial, aerial, and satellite air–ground integrated network (SAGIN) communication systems.

### 7.1. Terrestrial Communication

In hybrid FSO/RF scenarios, terrestrial networks are usually modeled as backbone networks with base stations (BS) connected between users, vehicles, and IoT on the earth’s surface. This network type faces two primary challenges, namely high-density areas and the Doppler effect. To overcome these issues, many researchers present solutions to improve system performance.

Sandeep et al. [111,112] introduce a V2I communication system in an HS hybrid FSO/RF system where the infrastructure operates as a DF relay. The model accounts for PE and the Doppler effect to evaluate OP and throughput performance [111]. Additionally, system delay and SEP performance are investigated in [112]. In the first connection phase, the data transfer occurs between the vehicular unit and infrastructure via an RF link, while in the second phase, the infrastructure connects with the BS via an FSO or RF link. The numerical results reveal a trade-off between OP and throughput. Also, the Doppler effect negatively affects OP performance. Moreover, [112] shows that increasing the FSO link distance can lead to a higher average end-to-end delay, but this effect can be mitigated by reducing the packet length.

**Figure 16:**
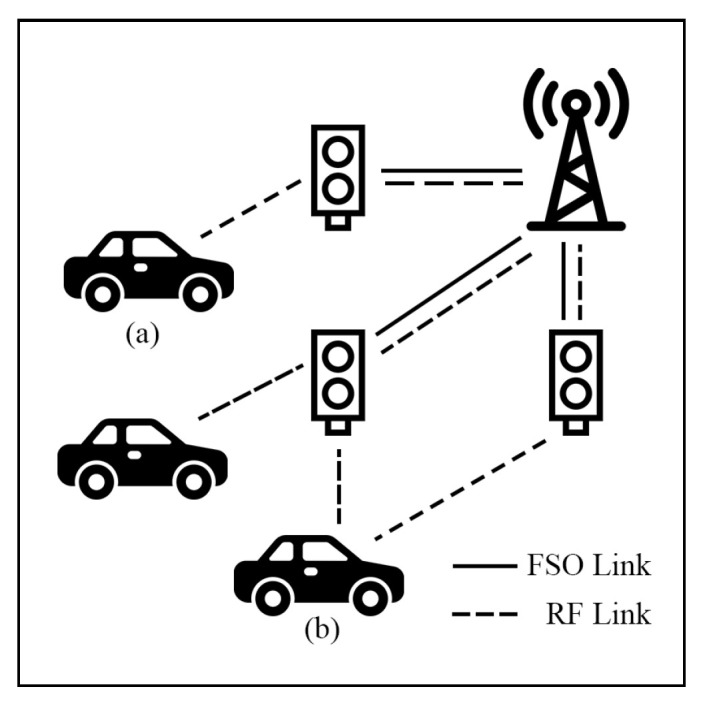
MIMO relaying hybrid FSO/RF model (**a**) SISO, (**b**) MIMO.

Payal et al. [113] developed a design of the V2I system by employing multiple relay units and multiple antennas at BS with an MRC scheme. In this system, vehicles connect to the nearest infrastructure, which can then hand over the data to other infrastructures, while the BS receives the data using the MRC scheme, as shown in Figure 16. The simulation results demonstrate the improvement of OP and throughput performance due to the use of multiple antennas with MRC techniques.

Rakia et al. [114,115,116] present a novel point-to-multi-point (P2MP) HS hybrid FSO/mmW system that links a central node with multiple remote nodes under GG/NM models. Each remote node is connected to the central node via a separate FSO link with a shared-RF backup link at the central node.

The authors [114] investigate a single remote node to analyze transmit buffer size, the efficiency of the queuing system, and RF link utilization performance. In [115], a throughput analysis is conducted. The studies assuming equal data rates for FSO and RF links demonstrate that incorporating the backup RF link significantly enhances system performance.

**Figure 17:**
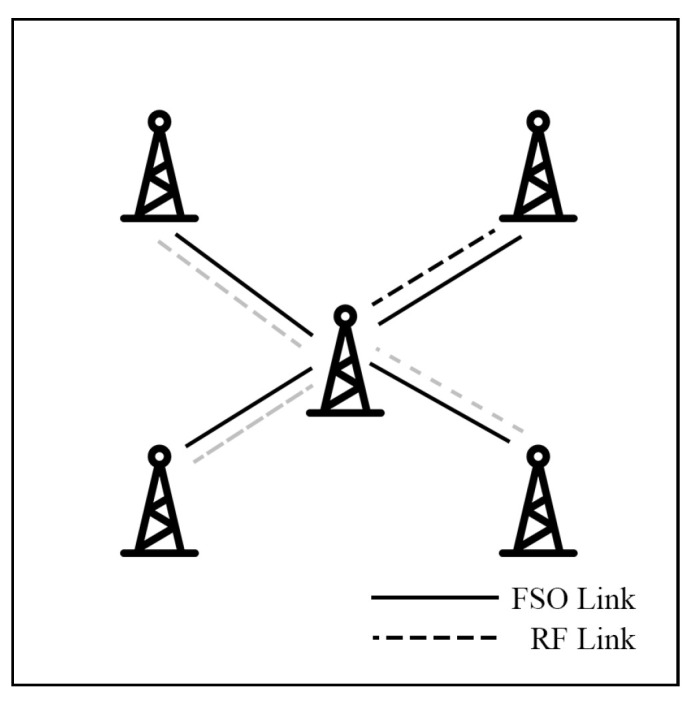
Hybrid FSO/mmW BS with Shared-RF frameworks.

The case of non-equal priority nodes is analyzed in [116]. The system introduces an algorithm to guarantee better QoS for high-priority users while still allowing lower-priority users to receive intended data, as shown in Figure 17. Additionally, the algorithm manages the trade-off between resource fairness and network throughput by adjusting the QoS in the persistence servicing protocol. Numerical results show that the system can ensure high-priority users receive better performance while low-priority users approach the intended throughput.

**Figure 18:**
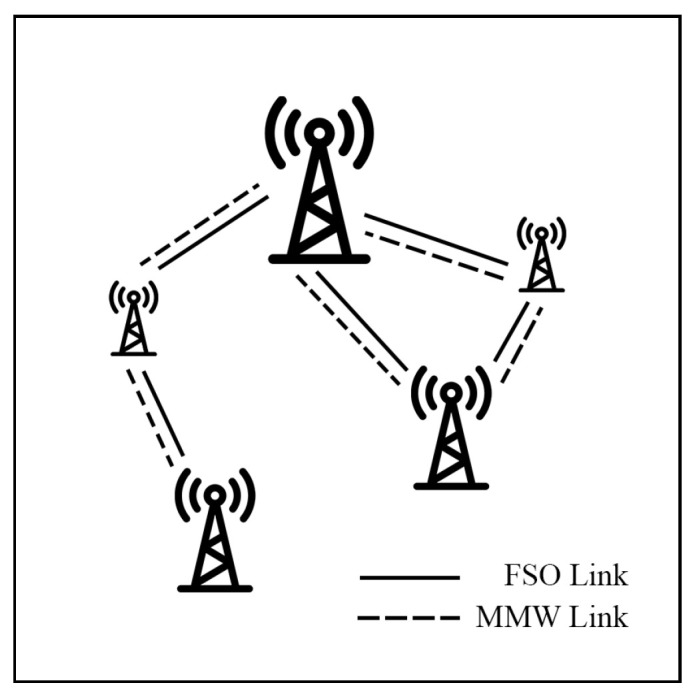
Hybrid FSO/mmW Base Station with Relay framework.

Sharma et al. [117,118,119,120] investigate a hybrid FSO/RF system with a DF relay for a backhaul network. Data can be transferred through a relay or directly through the BS, as illustrated in Figure 18. In [117], the selective link protocol with MRC is outlined to achieve high data rates and minimize OP performance. Additionally, SEP, OP performance, and the optimum switching threshold SNR value are evaluated under GG/NM models. However, the system calculations in this study do not account for PE. To address this limitation, [118] investigates the impact of PE and determines the optimal values for beam waist and receiver aperture size. Furthermore, to simplify the system and reduce costs, the SC scheme replaces MRC in [119], and the SC scheme with PE is further examined in [120] to determine OP and SEP performance. The models achieve better OP and SEP performance than conventional hybrid FSO/RF systems without MRC and SC schemes.

While the Doppler effect is mostly not considered in V2I systems, and handover scenario between FSO and RF at the base station remains understudied as well. These two aspects represent potential directions for future research. Moreover, the behavior differences between FSO and RF links, such as throughput, environment sensitivity, and reliability, should be carefully considered when designing hybrid communication frameworks.

### 7.2. Aerial Communication

Aerial communication systems, such as unmanned aerial vehicles (UAVs) and high-altitude platforms (HAPs), can extend coverage and mitigate NLoS issues in hybrid FSO/RF networks. HAPs, in particular, can reduce cloud attenuation by operating above cloud layers at altitudes of 17–22 km [121]. However, data transmission in these systems involves three stages: Air-to-Ground (AtG), Air-to-Air (AtA), and Ground-to-Air (GtA) links. These stages face challenges due to cloud interference and platform instability. Consequently, many researchers have investigated these systems to improve communication performance.

**Figure 19:**
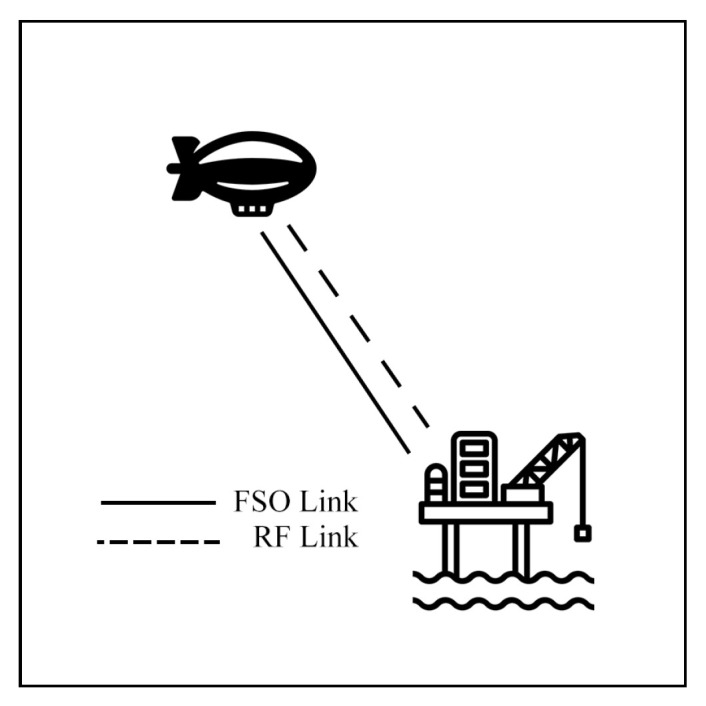
HAP-offshore hybrid FSO-mmW framework.

Starting with HAP schemes, Lyu et al. [122] investigate an HS hybrid FSO/RF system for HAP communication with offshore platforms, as shown in Figure 19. The simulation model analyzes switching probability under cloud fading, atmospheric turbulence fading in the GG model, and the offshore platform’s swing PE parameters. The results indicate that platform swing is a major factor affecting performance. Furthermore, the authors suggest an ATP system to mitigate the PE effect.

**Figure 20:**
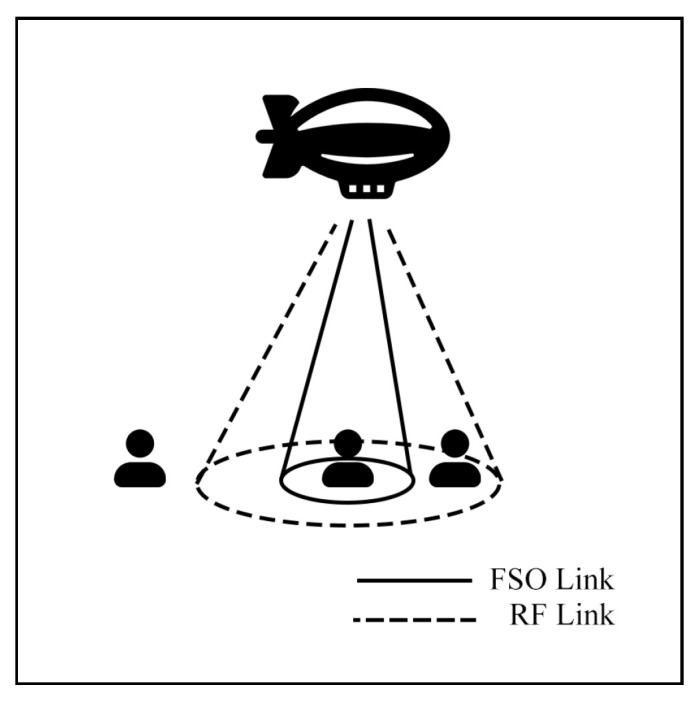
Ground-HAP hybrid FSO-mmW framework.

Tachikawa et al. [121] propose a Power Level Division Multiple-Access (PDMA) system for ground-to-HAP uplink networks with hybrid FSO-mmW, as shown in Figure 20. PDMA is a multiple-access technique in which the channel and power levels of each user are switched in every time slot to optimize throughput and user fairness. In comparison, the Non-Orthogonal Multiple-Access (NOMA) process transmits power differently in the same channel to ensure successful signal separation. The simulation results indicate that the PDMA system achieves a higher fairness index than the NOMA scheme, while the BER performance of PDMA is inferior to NOMA. Consequently, PDMA emerges as an alternative approach that offers high user fairness for hybrid FSO-mmW systems.

**Figure 21:**
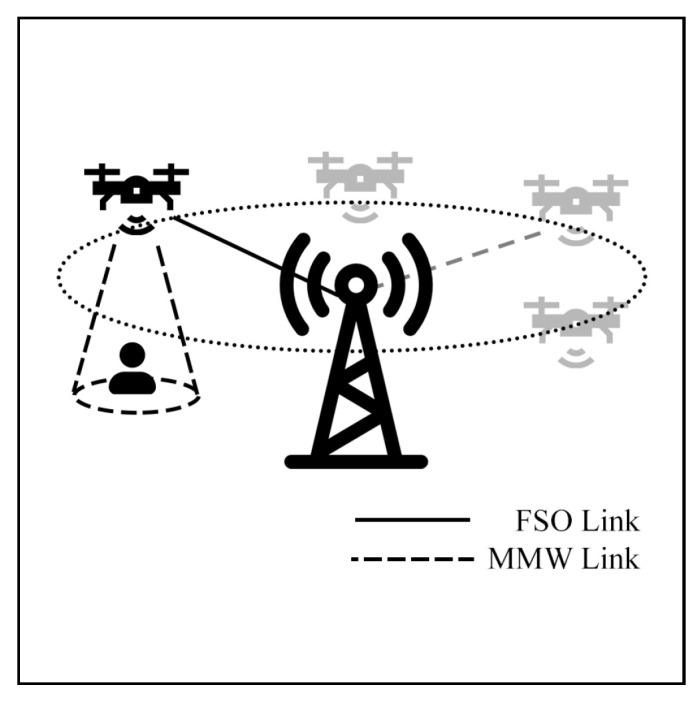
Movable BS UAV hybrid FSO/mmW framework.

From HAP shifting to UAV schemes, Usman et al. [123] demonstrate a hybrid backhaul system using FSO/mmW links for a UAV as movable BS. The UAV can establish a connection to fixed BS by FSO or mmW links. In the operation, when a UAV is moving, the mmW link is used, and then stationary hover, the FSO link is used as shown in Figure 21. Simulation results reveal that the UAV system outperforms the standalone RF or FSO connection in terms of overall BER during both stationary and flying periods.

**Figure 22:**
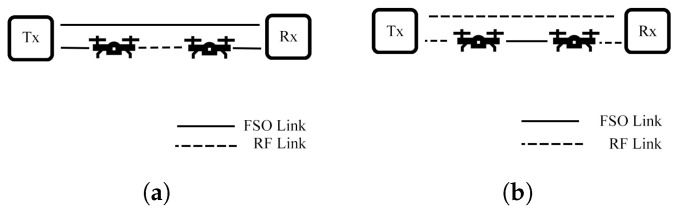
Hybrid FSO-mmW UAV relaying framework: (**a**) FSO as primary link, (**b**) RF as primary link.

Nafee et al. [124] designed a relay UAV-supported FSO link. The UAVs operate as movable relay FSO-mmW systems to intercept the FSO link during adverse weather conditions to ensure system reliability, as depicted in Figure 22a. Benchmark results based on Edinburgh and London weather statistics demonstrate that the approach can enhance OP and capacity performance. Moreover, Nafees et al. [125] designed a relay UAV-supported RF link. The design is almost the same, but now RF is the primary link, and FSO is the support link, as shown in Figure 22b. This design aims to increase BER and OP when rainfall occurs. They also added a reinforcement learning (RL) method to optimize UAV positions. The results indicate that the performance of the proposed system outperforms 117.05% of the standalone FSO or RF system.

Niu et al. [126] investigate a dynamic atmospheric-based topology reconstruction (DATR) model to mitigate the interruptions of FSO links and maintain reliability under changing weather conditions. There are two network reconstruction strategies: overall and partial network topology reconstruction. The system applies an improved conventional particle swarm optimization (PSO) method to achieve faster solution times. In simpler terms, the model reconstructs the communication path between UAVs to find the most stable route to maintain communication links. Numerous results show that the DATR with the PSO model can improve the connectivity and throughput compared with traditional models.

**Figure 23:**
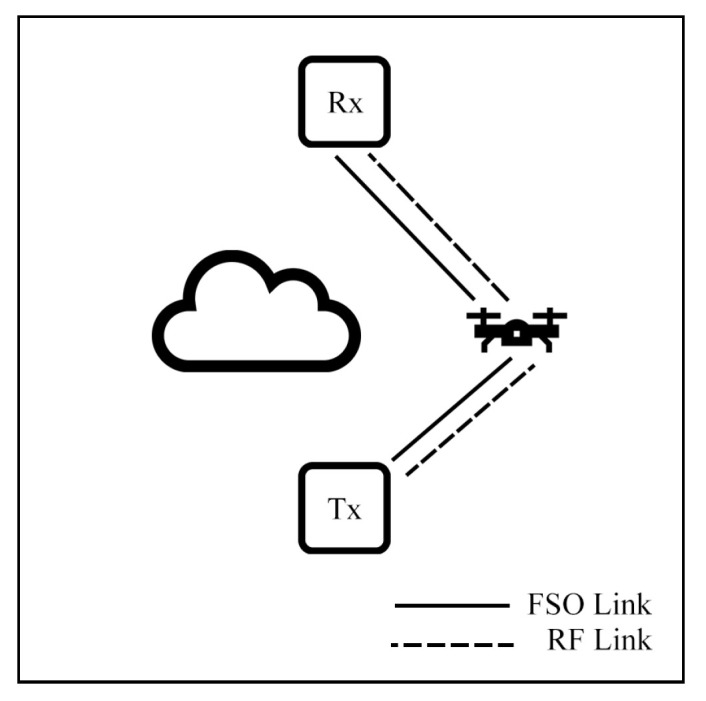
3D Trajectory Optimization for UAV-Assisted Hybrid FSO/RF Network.

Zhang et al. [127] optimized the UAV’s 3D trajectory to ensure LoS. An efficient Trajectory Planning Gaining-Sharing Knowledge (TPGSK) algorithm for UAV routes that avoid cloud obstructions was presented, as shown in Figure 23, and tested through numerical simulations. The algorithm shares data between the Tx, UAV, and Rx to create the route. Through this algorithm, the trajectories can maximize the data rate and minimize the UAV’s energy consumption.

**Figure 24:**
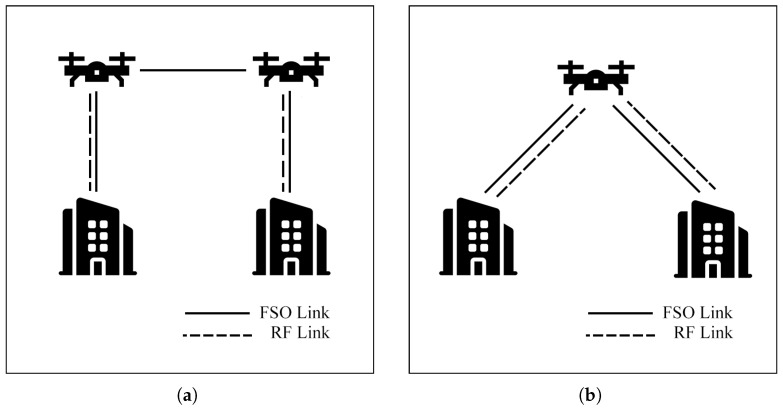
Hovering UAV-based hybrid FSO/RF system framework: (**a**) Two UAVs hovering, (**b**) One UAV hovering.

Singh and Swaminathan [32,128] propose a hovering UAV-based FSO communication system model. The system is divided into three parts: GtA, AtA, and AtG, as illustrated in Figure 24a. The AtA communication is examined in [128]. In the follow-up work [32], three comprehensive parts are analyzed. The system analysis is evaluated in terms of OP, SEP, and EC in diversity gain under various factors, including weather conditions, atmosphere models, PE impairment, beamwidth, FOV values, angle-of-arrival fluctuations, transmission power, link distance, and detection scheme. The numerical results display that the beamwidth, FOV, and transmit power significantly affect OP, SEP, and EC performance.

To simplify the system, a single hovering UAV is presented in [129], operating a UAV-based FSO system with a DF relay as illustrated in Figure 24b. The system is studied to understand its behavior and evaluate OP, SEP, and EC performance under different modulation techniques, weather events, beamwidth, FOV, and link lengths. The simulation results show that HD outperforms IM/DD. However, the UAV with a DF relay is inferior compared to a fixed station with a DF relay due to AoA fluctuations of the hovering UAV.

Zhang et al. [130] propose two UAV trajectory optimization schemes: slot-based and period-based. The slot-based approach optimizes data transmission in each time slot (for delay-sensitive data), while the period-based approach optimizes data over the entire flight period (for delay-insensitive data). Benchmark results demonstrate that the period-based scheme achieves better OP performance than the slot-based scheme.

Liu et al. [131] propose a deep reinforcement learning (DRL) method for UAV trajectory optimization in a hybrid FSO/RF system with a QoS guarantee. The study simulates a UAV hovering in urban environments under varying weather conditions. Proximal Policy Optimization (PPO) is a key system method for optimizing UAV trajectories by learning from machine-learning rewards. The results demonstrate the robustness and effectiveness of the algorithm for maintaining a reliable link with QoS via the UAV.

**Figure 25:**
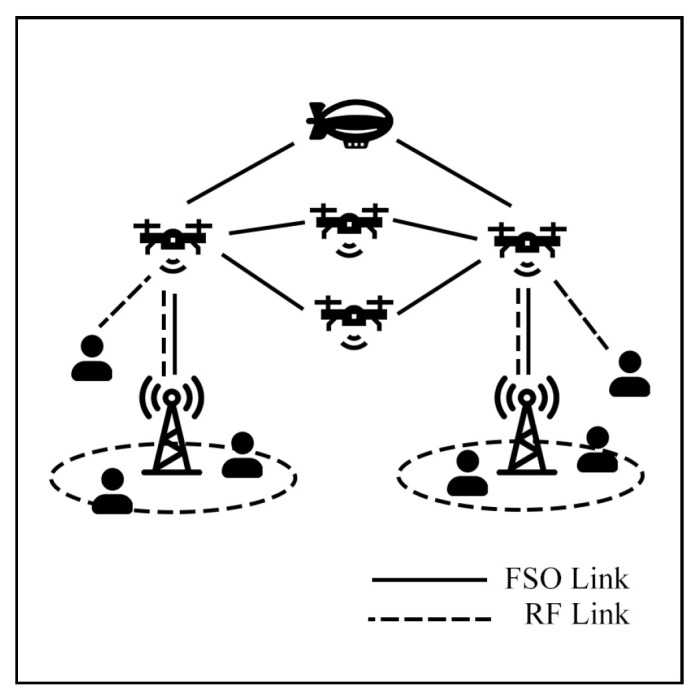
Aerial hybrid RF/FSO relay network framework.

Lastly, in comprehensive aerial networks, Erdogan et al. [132] analyze cognitive radio (CR) in the RF/FSO model to utilize the full frequency spectrum. This model integrates HAPs, UAVs, BSs, and user networks, as shown in Figure 25. The CR-assisted RF communication is proposed to deliver multi-user connectivity and optimize the utilization of the spectrum. The simulation under Loo’s model demonstrates that multiple HAPs and UAVs can serve as a backhaul and provide more reliability when using FSO and RF simultaneously. Also, they found that the CR overlay method is preferable to underlay and interweave methods in GtA communication.

As aforementioned, aerial hybrid FSO/RF systems often face platform instability, atmospheric disturbances, and vibration issues. However, these aspects have received little attention in the literature. Advanced APT systems may address the unstable issues, but further study is required. Additionally, operational power and time remain significant limitations of aerial platforms. Therefore, to address the power issues, the integration of FSO-SWIPT and RF-SWIPT represents a promising direction for future research [133].

### 7.3. Satellite Air–Ground Integrated Networks Communication (SAGIN)

Satellite communication (SATCOM) connects across global areas, covering most regions in the world. The satellites can be classified into three primary orbital types, including low Earth orbit (LEO), Medium Earth orbit (MEO), and geostationary orbit (GEO). LEO satellites are commonly used for communication applications due to their low latency and high bandwidth compared to other satellite types. On the other hand, the satellites have a limited field of view (FoV), which only connects to ground stations (GS) for short periods. As a result, to cover global connections, numerous LEO satellites are essential. Many researchers have proposed integrating LEO satellites into hybrid FSO/RF systems to enhance connectivity.

**Figure 26:**
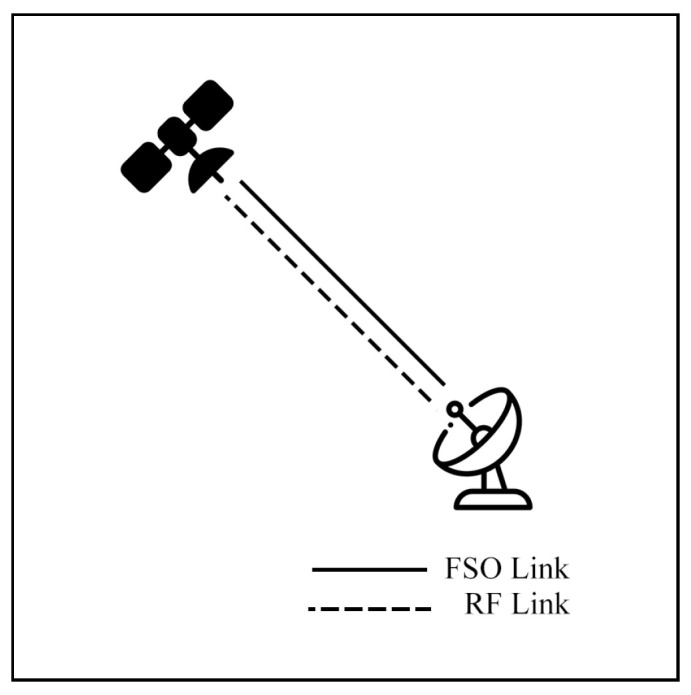
Satellite communication framework.

Li et al. [134] present a rate adaptation (M-QAM) hybrid FSO/RF link for SATCOM. A satellite is directly connected with GS via an FSO or RF link, as shown in Figure 26. The model is simulated to evaluate OP, BER, and throughput under GG and Rician fading models, considering PE and weather conditions. The results show that higher modulation orders can achieve better transmission rates under ideal conditions. On the contrary, in adverse weather, lower modulation orders perform better. Thus, the rate-adaptive design can flexibly switch the modulation order to achieve better performance across all climates.

Vishwakarma and Swaminathan [135] propose a direct-link SS hybrid FSO/RF system between a satellite and a GS. The system utilizes adaptive combining and MRC to examine EC performance under GG/κ-μ distribution models. The system transmits the signal over both FSO and RF links using adaptive combining with the MRC scheme to maximize the overall SNR). The results reveal that the system outperforms conventional hybrid systems, especially under wide zenith angles, severe PE, and high-velocity wind, in terms of EC performance.

**Figure 27:**
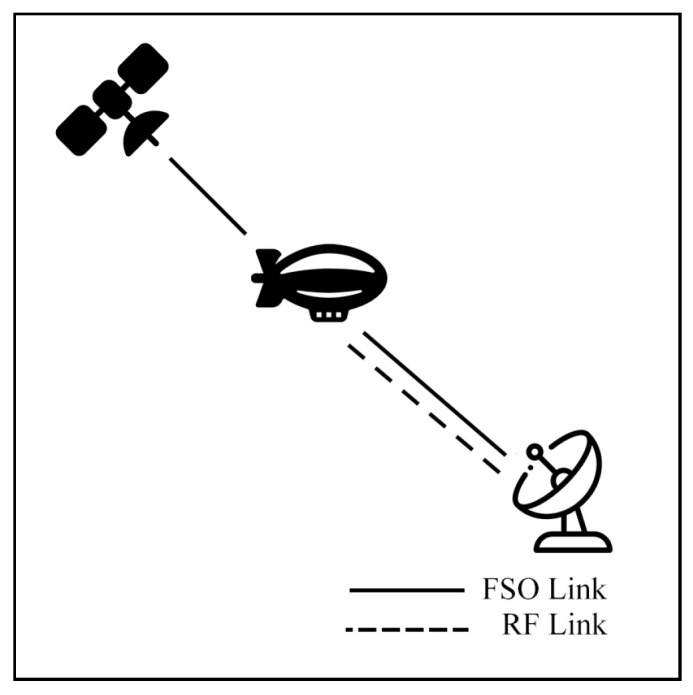
HAP relay-assisted hybrid FSO/RF SATCOM framework.

Swaminathan et al. [136] evaluate a HAP DF relay operating hybrid FSO/RF for the SATCOM as depicted in Figure 27. The system is simulated under the GG/Rician models to assess SEP and OP. Initially, the study investigates the potential of a downlink scenario. Then, a comprehensive end-to-end performance is presented [137]. The authors explore numerous diversity gains, namely IM/DD, HD schemes, PE effect, zenith angles, SNR threshold, SH, DH, wind speed, and transmit power. The numerical results [136,137] show that the HAP relay hybrid systems can enhance SEP and OP performance while the severity of atmospheric turbulence, PE, zenith angle, and wind velocity reduce overall performance.

Furthermore, Bithas et al. [138] introduce a new link selection method for the HS hybrid FSO/RF in SATCOM with a HAP relay. This method checks the state of the link at fixed time intervals, minimizing signal overhead, preventing the ping-pong effect, and improving reliability performance.

To further enhance OP performance, a multiple HAP backup relay links technique is suggested [73]. The results indicate that increasing the number of HAPs (*N* relays) improves OP. However, factors such as high-speed wind, zenith angle, and PE can negatively affect OP and SEP performance.

Shah et al. compare a direct link [135] with an indirect link [138] of hybrid FSO/RF using an adaptive-combining-based switching scheme, as shown in Figure 26 and Figure 27, respectively [139]. These systems are simulated under GG/Rician models. The results show that the indirect link, which utilizes HAP as a relay station, improves OP and SEP performance even under severe zenith angle, PE, and wind speed conditions. However, in high SNR regimes (e.g., very clear atmospheric conditions), the HAP relay does not contribute significantly to performance improvement.

Furthermore, Jain et al. [140] compare modulation techniques between OOK and PPM in the HAP relay-assisted scenario [135]. The results indicate that OOK provides higher reliability than PPM in strong turbulence, while PPM offers higher bandwidth in weak turbulence. Additionally, Jain et al. [141] evaluate Rayleigh and Rician distribution models, finding that the Rayleigh model is suitable for non-stationary HAPs, whereas the Rician model applies to stable HAPs.

**Figure 28:**
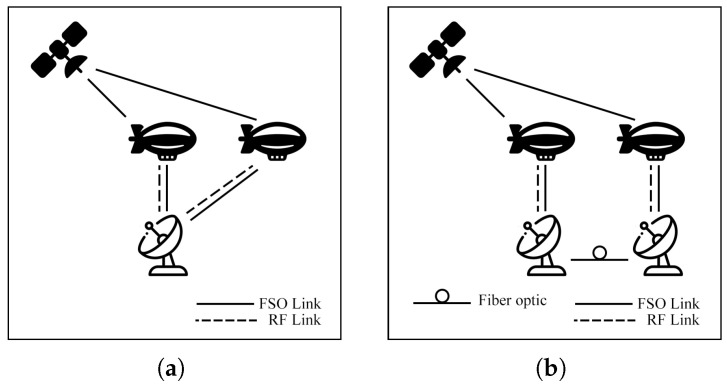
HAP-assisted hybrid FSO/RF SATCOM frameworks: (**a**) HAP diversity, (**b**) Site diversity.

In addition, Li and Li [142] investigate multiple HAP systems [73] and site diversity system-based hybrid FSO/RF systems in SAGIN as illustrated in Figure 28a and Figure 28b, respectively. They focus on the impact of RF interference and outdated CSI under GG/NM models. Numerical results show that increasing interference negatively affects OP performance, but using multiple antennas and applying diversity techniques can significantly improve the performance. The study concludes that while GS diversity offers better OP performance, it comes with higher complexity and cost compared to the multiple HAPs approach.

**Figure 29:**
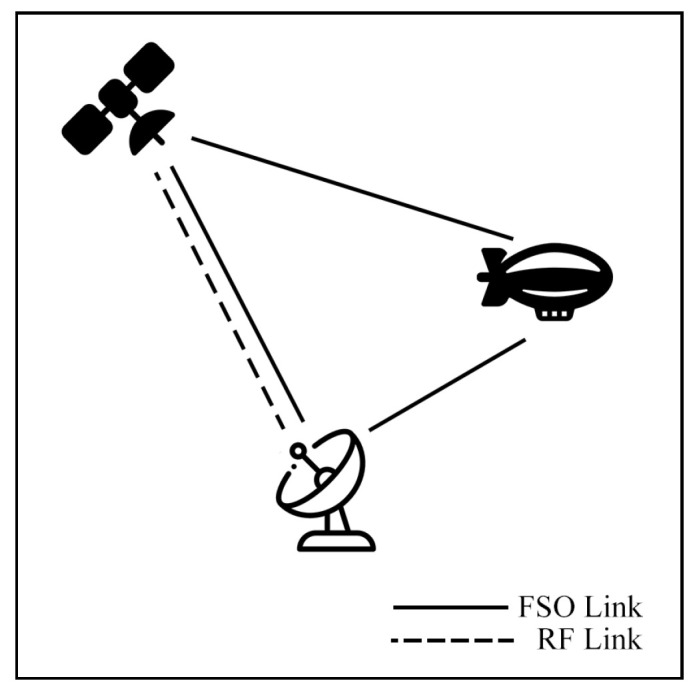
HAP relay backup link in hybrid FSO/RF SATCOM framework.

Samy et al. [143,144,145,146] introduce a hard switching (HS) hybrid FSO/RF SAGIN network that comprises a ground station and a HAP relay. The systems are simulated under GG/Rician models. The model uses the primary ground station to transfer data directly through a satellite via FSO or RF links, while another link combines with a HAP as a backup relay FSO link, as depicted in Figure 29. This design aims to mitigate atmospheric conditions to improve overall performance. In [143], the authors analyze the optimum switching threshold system and EC performance. Moreover, the satellite zenith angle is analyzed in [144]. In [145], the detailed design of the HAP relay system has been explained, along with an analysis of OP and throughput. Additionally, the EC performance is assessed in [147] under the effect of correlated turbulence in integrated SAG-FSO/SH-FSO/RF transmission for satellite communications. Furthermore, the SEP performance under different satellite zenith angles and PE is examined in [146]. The results in [143] show that IM/DD can achieve better EC performance over HD for all zenith angles. These findings contribute to a better understanding of how hybrid FSO/RF systems can enhance SAGIN communication performance in terms of OP, EC, SEP, and throughput under different conditions.

**Figure 30:**
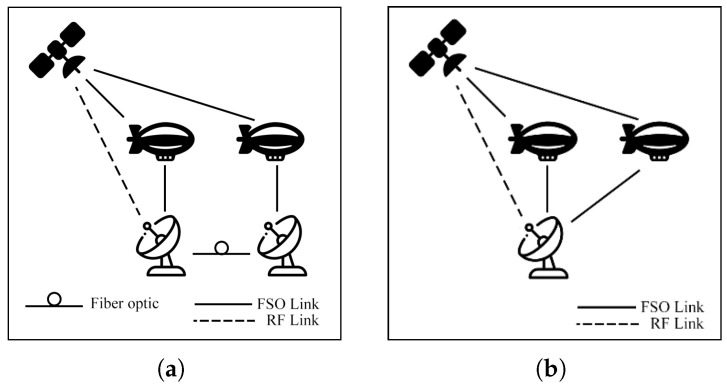
HAP relay-assisted SATCOM: (**a**) Site diversity framework, (**b**) HAP diversity framework.

In [148], the other two patterns, site diversity and multiple HAP relays, are mentioned. The site diversity framework can successfully mitigate atmospheric turbulence effects while the multiple HAP relays reduce cost and complexity, as shown in Figure 30. These frameworks can achieve EC of more than 3 bps/Hz. The design of multiple HAPs is further analyzed in [149]. Compared to site diversity, this framework shows many advantages, including lower transmission power, reduced implementation costs, and improved SEP performance. These simulation results confirm that the diversity strategy can further reduce the impact of climate, resulting in better outage performance.

**Figure 31:**
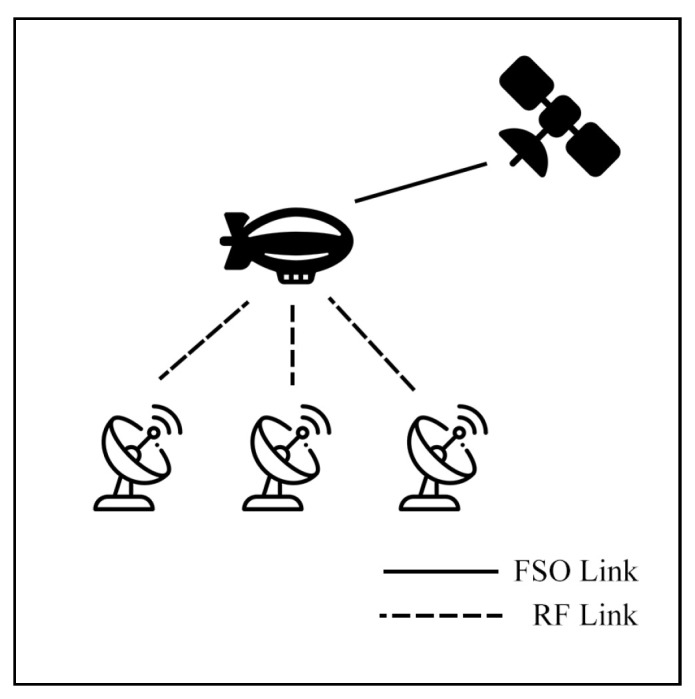
HAP relay-assisted SATCOM framework.

Hariq and Seshadri [150] proposed MIMO RF ground station and FSO HAPs for satellite links, as shown in Figure 31. The study identifies the minimum antenna spacing for the ground station to maximize the capacity. The equations were derived through log-normal and MIMO models under various antenna spacings. The simulation results show that the MIMO RF link with spatial multiplexing technique can significantly improve capacity between the ground station and HAPS.

**Figure 32:**
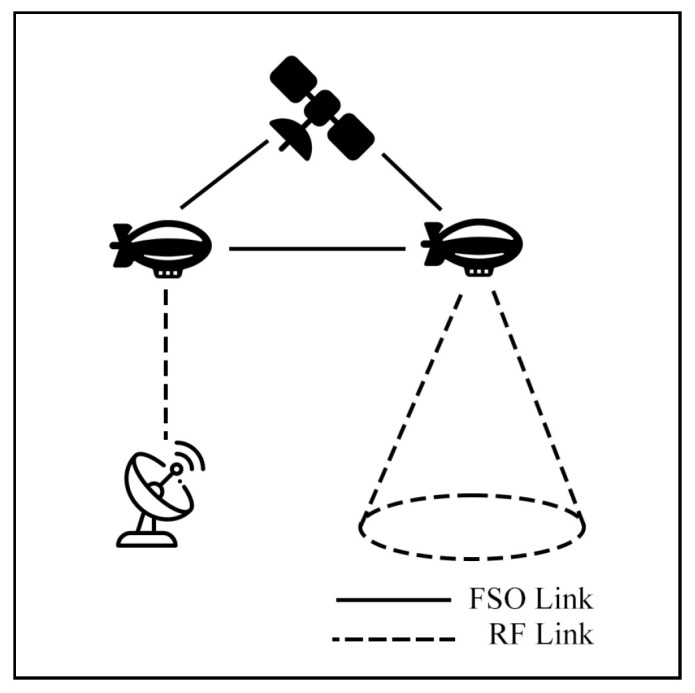
HAPS-assisted hybrid RF-FSO framework.

Yahia et al. [151] propose HAPS-assisted hybrid RF-FSO multicast communications. There are two scenarios: one using HAPS systems alone and another incorporating a LEO satellite to address the NLoS issue between two HAPs, as shown in Figure 32. The study assesses OP, ER, EC, and energy efficiency performance under Weibull/Rician models with the impact of beam wander, PE, aperture size, zenith angle, and wind speed. The results indicate that the satellite-assisted scenario enhances OP while HAPS-assisted achieves better energy efficiency.

**Figure 33:**
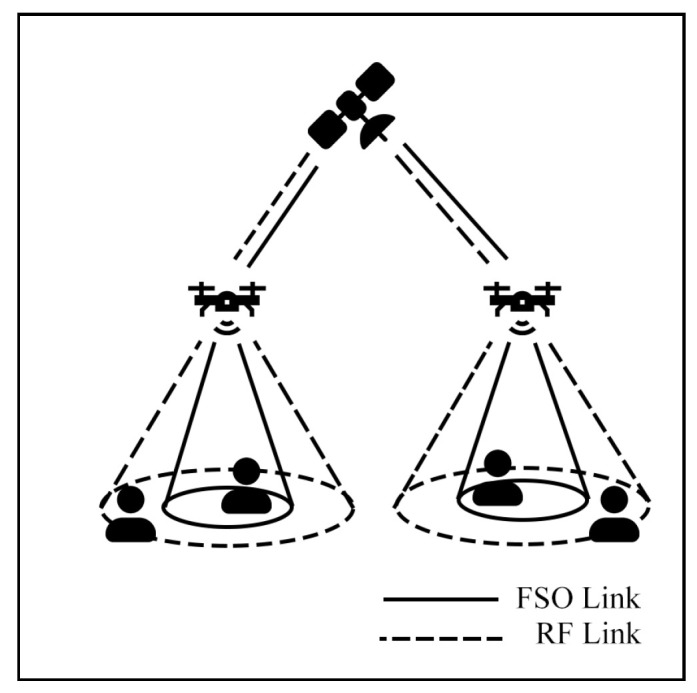
Multiple UAVs hybrid FSO/RF SATCOM framework.

Shifting from HAP to UAV, Dang et al. [152] combine rate adaptation-aided data frame allocation design with HS hybrid FSO/RF satellite-assisted where multiple UAVs serve as flying base stations, as shown in Figure 33. This design adaptively adjusted data frames based on the channel conditions of UAVs to meet QoS requirements and ensure latency fairness among the UAVs. The study simulates under Japan networks. The system can reach a maximum throughput of 1.9 Gbps and sustain a throughput above 800 Mbps for 170 s. These results show the potential model to enhance network performance in practical scenarios.

**Figure 34:**
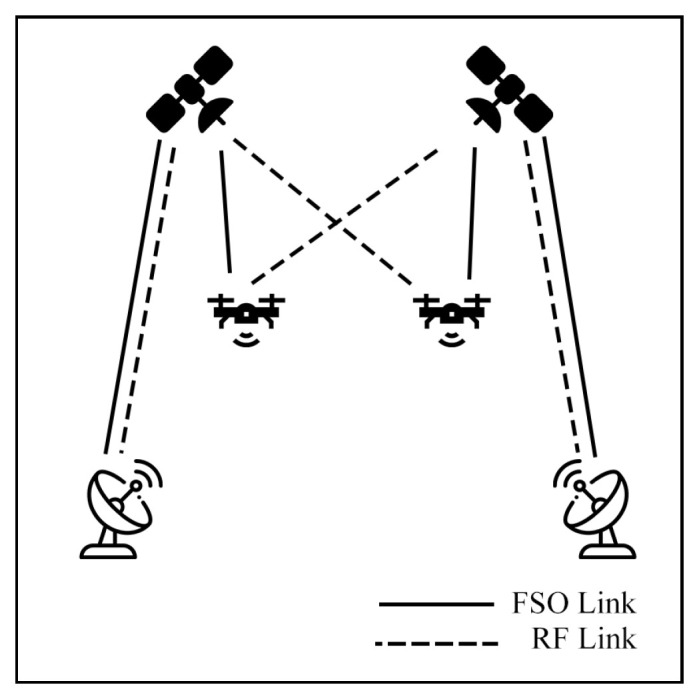
UAV relay-assisted SATCOM framework.

Lee et al. [153] simulate a satellite-UAV integrated hybrid FSO/RF with a multi-agent deep reinforcement learning (MARL) framework. The network consists of LEO satellites and UAVs that act as relay units between the satellites, as depicted in Figure 34. The UAVs optimize their locations and determine the associations with satellites and ground terminals in real time by the MARL algorithm to maximize throughput and energy efficiency. The MARL algorithm maps the source, destination, and relay locations to learn and provide the UAV flight route that maximizes both throughput and energy efficiency. The mathematical simulation shows the significance of UAV trajectory management in enabling high-throughput communication. Besides, the framework demonstrates a remarkable increase in peak throughput by a factor of 62.56 and a significant improvement in worst-case throughput by a factor of 21.09, outstanding scenarios relying on RF or FSO links only. However, compared to an inter-satellite link, this scheme is less effective.

**Figure 35:**
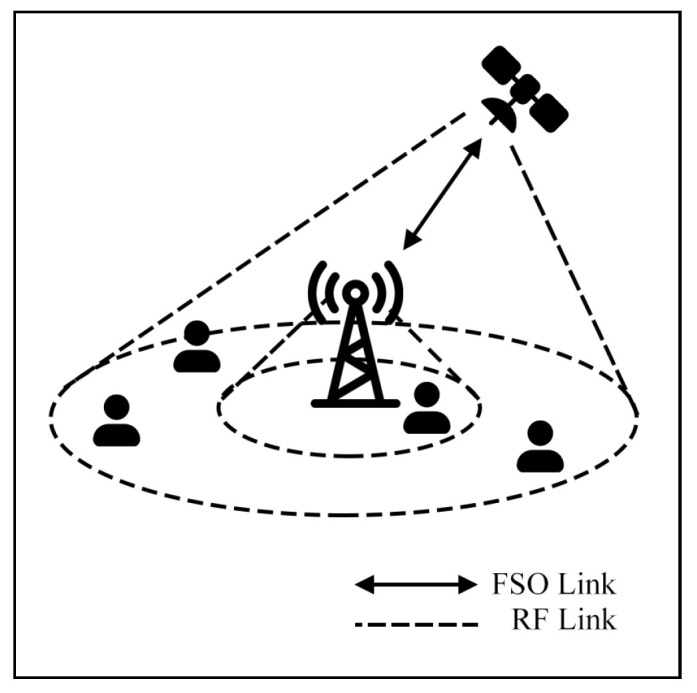
Parallel FSO-RF SATCOM transmission framework.

Samy et al. [154] presented a downlink satellite that can transfer data via FSO-RF with and without BS in different coverage areas simultaneously. In the system, a BS, as a relay, receives the FSO signal from a satellite and distributes it to users, as shown in Figure 35. The satellite also sends data via RF to users directly to increase coverage areas. The model shows that users can reach 2.2 Gbps at high-traffic demand via the FSO-RF link while maintaining extensive coverage via the RF link.

**Figure 36:**
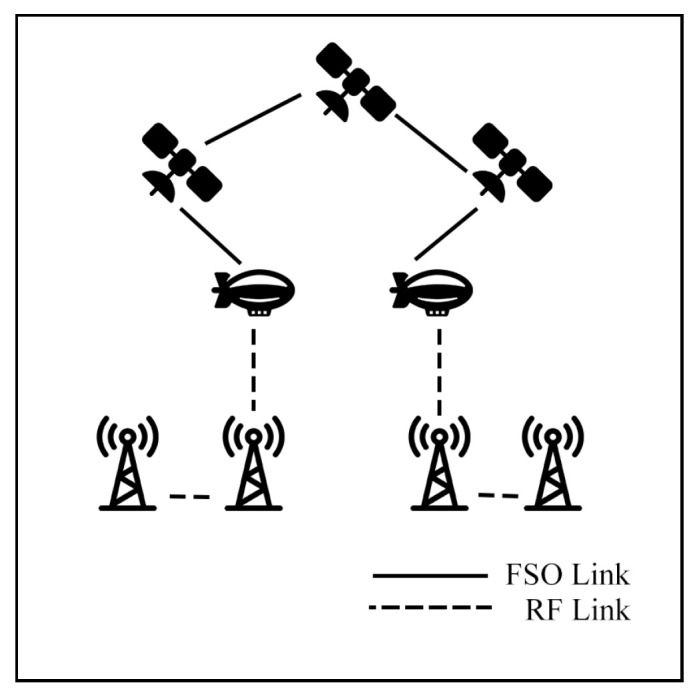
FSO/RF satellite HAP-ground system framework.

Guo et al. [155] propose an intelligent routing strategy for a hybrid FSO-RF system while ensuring QoS using a deep reinforcement learning (DRL) algorithm. Initially, the system integrates ground stations (GSs), high-altitude platforms (HAPs), and satellites (SATs), with HAPs acting as relays that receive RF signals from GSs and transmit the signal to SATs via FSO links, as shown in Figure 36. In subsequent published paper [156], the system is refined by incorporating more complex network elements such as users and inter-ground links. DRL training optimizes routing and energy efficiency through state, action, transition, and reward states. Simulation results demonstrate the algorithm’s effectiveness in selecting dynamic routing for time-varying link conditions while reducing energy consumption.

Nguyen et al. [157,158] proposed an order adaptive (M-QAM) design for hybrid FSO/RF with HAP AF relay. The system [157] connects a GS to a satellite through a HAP, using either FSO or RF links between the GS and HAP, while the HAP connects to the satellite via an FSO link only. In [158], a UAV capable of operating both FSO and RF links is introduced. Through their results, an adaptive rate system design provides better throughput, OP, and BER performance compared to a fixed-rate design under various factors, including beam loss, cloud attenuation, PE, and weather variability. Additionally, the UAV further improves system performance.

**Figure 37:**
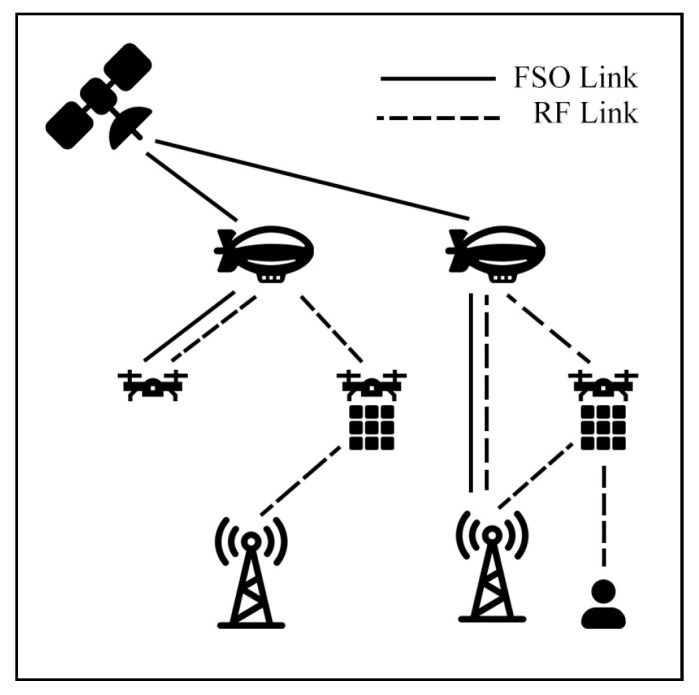
HAP-assisted satellite network with UAVs framework.

In [159], an advanced design of the order adaptive HS hybrid system is proposed, where a UAV equipped with an IRS is deployed. This system integrates a HAP that directly connects to a satellite via an FSO link and has two connections to a GS: a direct FSO link and an indirect IRS-UAV relay FSO link. The indirect relay FSO link is activated when the direct link between the HAP and GS degrades to improve link availability, as shown in Figure 37. Moreover, Li et al. [160] analyze the system [159], specifically considering the impact of HAP relay links on OP, BER, and EC performance. An asymptotic analysis of OP and BER provides deeper insights into the numerical model. The simulations reveal that higher UAV altitudes (near the HAP flying levels) achieve better data rates when an IRS is used to focus the UAV-GS beam, compensating for beam divergence and maintaining signal quality. Additionally, the proposed system demonstrates significant performance improvements in terms of OP, transmission rate, and EC under challenging environmental conditions.

Guo et al. [161] analyze an uplink IRS-UAV with a HAP system [159] using machine-learning (ML) techniques. They employ an asymmetric long short-term memory (LSTM) combined with a Deep Deterministic Policy Gradient (DDPG) algorithm to optimize beamforming and phase shifts. Wu et al. [162] further explore and explain the system in more detail, showing that the ML-based system outperforms the system without ML techniques in terms of EC performance. In the following year, Guo et al. [163] improved the IRS-UAV with a HAP system model by employing a rate-splitting multiple-access (RSMA) strategy and an access-free federated deep reinforcement learning (AFDRL) framework. The UAVs were trained to find optimal flight trajectories that maximize energy efficiency. The results indicate that the proposed system saves 7% more energy compared to the AO scheme [161].

In conclusion, SAGIN represents a crucial advancement in hybrid FSO/RF communication, offering worldwide connectivity. Numerous publications highlight the seamless integration of LEO satellites, HAPs, UAVs, and GSs. The use of adaptive modulation, diversity strategies, relaying, and machine-learning algorithms further enhances the system’s efficiency. As a result, hybrid FSO/RF SAGIN has become a strong candidate for future B5G communication networks. However, the lack of integration between global communication systems such as terrestrial networks and SATCOM remains insufficiently examined, as most literature focuses primarily on satellite communication.

### 7.4. Other Approaches

Nguyen et al. [164] investigate the TCP variants for HS hybrid FSO/RF in satellite fading channels under GG and NM models. The study focuses on a throughput analysis of the TCP protocols, including TCP NewReno, Hybla, Cubic, and High-speed TCP (HSTCP). The numerical results reveal that TCP Cubic outperforms the other four TCP protocols in SATCOM networks under the impact of transmission and congestion loss.

The security aspects of hybrid FSO/RF systems are studied. In hybrid systems, each RF and FSO link presents unique vulnerabilities. RF links are more susceptible due to the broadcasting nature, while FSO links can suffer from the PE effect and eavesdropper relays. In [165,166,167], closed-form expressions for the secrecy outage probability of RF-FSO systems under terrestrial and SAGIN are presented. The man-in-the-middle attack at relay stations and the impact of aperture averaging and antenna correlation are analyzed [168]. Other studies have focused on specific challenges in NOMA networks to optimize secrecy [169].

Finally, antenna design for hybrid FSO/RF systems is a key area of research. Studies on microstrip antennas are highlighted in [64], and comparisons of antenna materials for hybrid systems are presented in [170]. Outdoor experiments confirm the effectiveness of these antennas [171]. Additionally, dual-purpose antennas are developed to support both FSO and RF technologies [172,173]. These developments mark a significant advancement in antenna design for hybrid FSO/RF networks.

## 8. Practical Implementations

In this section, practical experiments of hybrid FSO/RF systems are explored. Unlike the previous sections, which focus on mathematical equations and simulation analysis, this section is grounded in real-world implementations that assess the performance under various atmospheric conditions and mechanical factors. These setups validate the theoretical models and show the potential of hybrid systems. Two main experimental categories are highlighted: atmospheric mechanics and machine-learning-based models.

### Atmospheric Effects Experiment

Atmospheric conditions are a serious factor in the performance of hybrid FSO/RF systems, particularly influencing the switching mechanism. Therefore, it is important to understand weather dynamics and test in real-world conditions.

**Figure 38:**
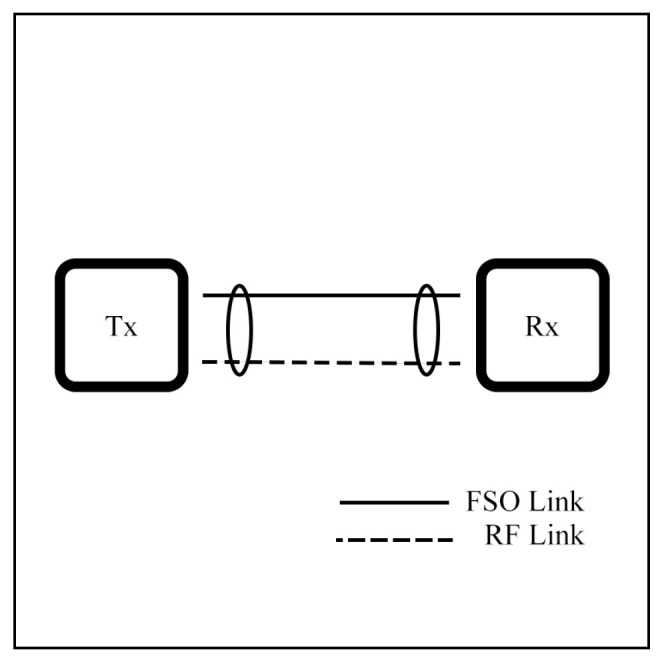
Hybrid FSO/RF in Ethernet link aggregation experiment.

Chen [59] conducted an HS hybrid FSO/RF experiment using ethernet link aggregation on a layer 2 switch. This system includes OptiX SpaceLink400 (Huawei Technologies Co. Ltd., Guangdong, China) for FSO system, RF modules, and Ethernet switches to combine FSO and RF links, as shown in Figure 38. The results indicated that the hybrid system’s bandwidth was slightly lower than a single FSO channel due to processing overhead in the Ethernet switch’s channel-sharing mechanism (In simpler terms, FSO + RF + overhead). Nonetheless, this approach improves reliability and utilizes both channel capacities effectively.

**Figure 39:**
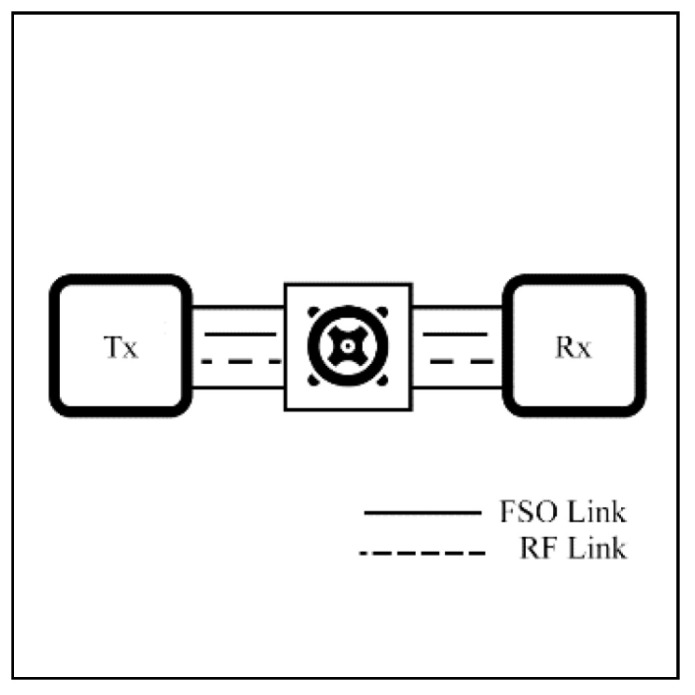
Experimental setup for the hybrid FSO/RF link.

Esmail et al. [60] experimented to assess the performance of parallel hybrid FSO/RF under dust storm conditions. The setup included a TeraXion laser diode (TeraXion Inc., Quebec, Canada), a Finisar photodetector (Finisar Corporation, Sunnyvale, CA, USA) for the FSO module, and an SHF807 amplifier (SHF Communication Technologies AG, Berlin, Germany) for RF module, and a controlled environment chamber, as shown in Figure 39. To investigate the effect of dust storms, sample dust was collected from the area surrounding Riyadh city. The results revealed that under visibility below 200 m, the FSO’s link performance declined significantly while the RF signal remained stable. This study demonstrates that a hybrid FSO/RF system effectively mitigates the impact of dust storms, with the RF link maintaining connectivity during severe dust environments and the FSO link enhancing system throughput under clear conditions.

These are good examples of hybrid FSO/RF systems in a real-world implementation. However, the comparison between mathematical models and practical implementations remains underexplored. Additionally, protocols for hybrid FSO/RF systems still have significant room for development, as current studies have primarily focused on Link Aggregation Control Protocol (LACP). Other load-balancing protocols such as Hot Standby Router Protocol (HSRP), Least connection load-balancing algorithm, and Weighted Round Robin load-balancing algorithm [174] require further investigation. Moreover, the studies have been conducted in controlled chamber environments rather than real-world settings, which are more dynamic. This represents a significant gap in real-world scenarios that need to be addressed in the hybrid system research.

## 9. Discussion

This section explores the key findings from theoretical models and practical implementations. It is obvious that while hybrid systems offer a promising solution to address the individual weaknesses of RF and FSO technologies, several challenges remain. These challenges are tied to the dynamic nature of events, system complexity, and adaptive mechanisms. The following section will discuss open challenges identified in the literature, such as interference mitigation, throughput enhancement, and machine-learning algorithms. This discussion will also lead to the potential roadmap for future research, areas that need further exploration and development.

### 9.1. Open Challenges

Based on the research works, these are important challenges that we can identify regarding hybrid FSO/RF.

Implementing in sensors and IoT networks requires more research. Many devices only support RF technologies, while FSO is slightly supported. The lack of standardized FSO and the hybrid network for the hardware and protocols presents an open challenge. Although the IEC 60825-1 standard [175] provides a classification for laser safety, it primarily limits the permissible optical power used in communication and does not address implementation or adaptation for sensor technologies.

Environmental sensitivity and channel conditions are the most significant challenges. FSO links are highly susceptible to climates such as fog and dust, which can degrade the signal. Also, atmospheric turbulence, which reduces signal strength and introduces phase distortions, is a critical factor. However, RF links face spectrum interference and congestion. Developing efficient algorithms that predict or monitor weather remains an open issue.

A seamless switching mechanism is required for hybrid systems. Sophisticated algorithms that hand over between FSO and RF during transitions are important for ensuring system connectivity. Implementing an efficient switching mechanism that minimizes negative effects and prevents ping-pong effects remains an ongoing challenge.

Network selection criteria are a complex decision-making process. Various factors, such as data rate requirements, QoS, feedback data, SNR threshold, and power constraints, are involved. While many simulations focus on optimizing the SNR threshold, real-world implementations often rely on RSSI instead. These criteria require further refinement to ensure optimal system performance.

Managing interference and noise in hybrid FSO/RF systems is especially challenging in environments where multiple systems coexist. In situations like dense urban areas, where RF congestion is common and adverse weather conditions amplify noise on hybrid networks, more advanced mitigation techniques are required to reduce the negative effects on communication quality.

Performance enhancement is key to developing the system. While these hybrid FSO/RF systems are designed to compensate for each technology’s weaknesses, other performance aspects, such as throughput, QoS, latency, and user fairness, also need further attention. Balancing these factors, resource efficiency, and system complexity for specific applications needs further investigation.

Integrating UAVs, HAPs, IRS, BSs, and satellites as relay nodes add complexity to hybrid FSO/RF systems. Major challenges include PE impairment, zenith angle variation, Doppler effects, and platform instability. Despite these challenges, these platforms can improve overall performance. Therefore, developing solutions to address these issues is essential.

The lack of real-world implementations is another issue for hybrid FSO/RF. Most publications rely on simulation and mathematical models, which are effective in controlled environments. However, practical testing in diverse and uncontrollable environments is important. The challenges of deploying these systems include the high cost of infrastructure and technical limitations, which remain critical challenges.

Machine Learning (ML) offers a promising solution to enhance decision-making processes. However, ML requires high-quality data and suitable algorithms to achieve accurate predictive models. Ensuring fast computation to respond to rapid environmental changes is crucial. Therefore, ML models need continuous refinement and retraining to adapt to changing conditions over time, posing an ongoing challenge in dynamic environments.

### 9.2. Future Research Directions

The following outlines future directions that have the potential to be deployed in hybrid FSO/RF systems. These areas focus on improving adaptability, performance, and integration with emerging technologies to meet growing communication demands.

Weather monitoring and prediction can be a main area of future research. The reliability of CSI, which transfers data feedback to monitor the transmission route, is a crucial part. Techniques such as acknowledgment signals, automatic repeat requests, error-detecting codes, and even systems that operate without CSI could be explored further. Additionally, prediction mechanisms that relay metrics in terms of RSSI parameters to forecast weather patterns via weather prediction models will be important directions for future research.

The switching mechanism between HS and SS is a mandatory technique. However, the integration of network management techniques such as load-balancing and Software-Defined WAN (SDN) remains underexplored. These can be candidates to handle the switching mechanism.

Resource allocation strategies require further research. This involves not only power and bandwidth allocation but also ensuring fair resource distribution among users while maintaining system performance during handoffs between FSO and RF. Balancing computational complexity with real-time decision-making will also be a key challenge.

Advanced network selection algorithms beyond traditional SNR and RSSI metrics are needed. Future studies could investigate the combination of intelligent switches with coding to make decisions based on multiple criteria, such as latency, user priority, bandwidth allocation, environmental conditions, and application-specific requirements. For example, game theory models have been proposed to optimize decision-making processes where multiple users and applications compete for network resources.

Adaptive schemes require further optimization. With changing environmental and channel conditions, systems must dynamically adapt techniques and transition smoothly between FSO and RF links. Both adaptive modulation and adaptive power approaches can adjust system configurations to improve resilience and dynamically respond to fluctuating weather and environmental conditions.

Cognitive techniques are another promising approach. These techniques use intelligent processes that dynamically adapt configurations based on changing environments. For instance, cognitive radio enables spectrum sensing, management, and sharing, while cognitive networks optimize routing and QoS in real time.

ML into hybrid systems is an emerging solution. This includes using ML for adaptive switching decisions, optimizing modulation schemes, enhancing resource allocation strategies, and forecasting weather events. For example, DRL is particularly effective in handling complex tasks, managing resources, and predicting environmental changes, making it well-suited for development in hybrid systems. Additionally, the multi-criteria decision-making and RL are being explored for more refined decision-making processes.

Finally, the potential for quantum communication and FSO 10 μm technology to integrate with hybrid FSO/RF is a necessary area for study. Quantum techniques such as quantum key distribution can ensure security over FSO links [176], while 10 μm technology can provide better tolerance in a limited atmosphere [177]. Researching these technologies within hybrid systems could lead to breakthroughs in data communications.

## 10. Conclusions

Hybrid FSO/RF systems are proposed to exploit the strengths of both FSO and RF communication technologies. FSO systems offer high bandwidth and enhanced security but are sensitive to atmospheric turbulence and conditions. In contrast, RF systems provide more reliability during adverse weather and, nevertheless, suffer from bandwidth limitations and interference issues. This review has discussed key advancements in hybrid systems, including the switching mechanism and the integration across ground and space platforms. Techniques such as cognitive radio, adaptive modulation, encoding, MIMO, IRS, and ML have been deployed to improve system performance. Additionally, practical implementations, including field tests and chamber tests, were explored.

Sensor devices continue to expand and support as a backbone of smart applications. Providing a high-speed and low-latency network is crucial to supporting real-time applications. Hybrid FSO/RF systems have the potential to address the growing demands of sensor applications by offering high capacity, low latency, and adaptive communication solutions. Therefore, to fully unlock the hybrid FSO/RF network potential, future research should focus on advanced networking strategies, optimized resource allocation, energy-efficient designs, intelligent network management, adaptive transmission schemes, protocols, and cognitive systems. These improvements will enhance the system to support next-generation sensor applications, ensuring robust and high-performance connectivity in real-time conditions.

## Figures and Tables

**Table 1:** Comparison between FSO and RF Technologies.

Properties	RF	FSO
Wavelength [31]	1 mm–100 m	10 nm–1600 nm
Spectrum regulation [16]	Yes	No
Transmission and receiver [38]	Antenna	LD/LED and PD
Noise [37]	Electromagnetic field	Ambient light
Main propagation effect [16]	Attenuation, fading	Attenuation, turbulence
Obstacle Penetration [30]	High	None
Environmental resilience [30]	High	Low–medium
Data Rates [16]	Mbps–Gbps	Gbps–Tbps
Safety [39]	Electromagnetic field radiations	Eye and skin-safe
Security [16]	Moderate to high	High
Cost [19]	Generally low	Moderate to high
Deployment Flexibility [16]	High	Moderate (LoS needed)
Reliability (various conditions) [37]	High	Moderate

**Table 3:** Summary of hybrid RF/FSO switching mechanisms in past studies.

Category	Reference	Objective	Simulation Models	Contribution
Hard Switching	Kirubakaran & Selvaraj [46]	Analyze an adaptive order M-PSK modulation performance	GG/κ-μ	Provides closed-form expressions for OP, ASER, and RF interference.
	Vishwakarma & Swaminathan [76]	Investigate adaptive modulation performance	Malaga/α-η-κ-μ with PE	Derives asymptotic SEP expression.
	Nath et al. [77]	Evaluate BPSK modulation in a shared-RF scheme	GG/NM	Presents closed-form OP and BER expressions for cognitive RF.
	Nath et al. [78]	Examine a cognitive RF system in the shared-RF model	GG/NM	Highlights cognitive RF performance improvements.
	Nath et al. [79]	Compare shared RF and on-demand RF schemes	GG/NM with PE	Identifies optimal rate and power adaptation and validates benefits of cognitive RF.
Soft Switching	Shrivastava et al. [70]	New switching scheme proposal	Negative Exp./Rayleigh	Introduces a shared-RF and On-demand RF system.
	Vishwakarma & Swaminathan [56]	Analyze the EC performance in the hybrid system with adaptive switching model	Malaga/κ-μ with PE	Optimize the optimum switching threshold SNR and beamwidth values for the best EC performance
	Gupta et al. [52]	Investigate an adaptive combining hybrid system with HD and IM/DD techniques	F-distribution/NM	HD is more reliable than IM/DD in every turbulence conditions
	Mehta & Sengar [81]	Simulate OP equations for modified-switching system	GG/Exponential	Optimize SNR threshold values for the system to increase reliability
	Roumelas et al. [44]	Demonstrates a triple hybrid FSO/RF/mmW system	Negative exponential/Rayleigh/Weibull	The triple hybrid system can achieve $10^{-11}$ OP probability.
Relay	Sun et al. [83]	Evaluate AF/DF relaying in hybrid FSO/RF under IM/DD	GG/α-F with PE	Derives the asymptotic expression of OP, BER, capacity, and EC performance.
	Ninos et al. [41]	Investigate a full-duplex relaying with parallel hybrid systems under RSI, IQI	GG/NM with PE	Derives the asymptotic expression of OP in a high SNR regime.
	Bag et al. [45,74]	Proposed hybrid systems with DF relaying.	GG/Rayleigh	The system improves reliability performance via relay switching.
	Liang et al. [91]	Evaluate a DF relaying hybrid FSO/RF-mmW system	Log-Normal fading/NM	Confirm the DF relay can improve system reliability
	Sharma et al. [86]	Analysis of IRS-assisted hybrid FSO/RF system	GG/Rayleigh with PE	the IRS-assisted systems enhance BER and EC performance
	Verma et al. [87]	Present a dual-hop DF relay and IRS system with H-ARQ	GG-Rician and Rayleigh with PE	the protocols and IRS can compensate atmosphere effects

## Abbreviations

The following abbreviations are used in this manuscript:

AF	Amplify and Forward
ATP	Acquisition, Tracking, and Pointing
BER	Bit Error Rate
BS	Base Station
CDF	Cumulative Distribution Function
CSI	Channel State Information
DF	Decode and Forward
EC	Ergodic Capacity
FSO	Free-Space Optical Communication
GG	Gamma–Gamma Model
GS	Ground Station
HAP	High-Altitude Platform
HD	Heterodyne Detection
HS	Hard Switching
IM/DD	Intensity Modulation/Direct Detection
IoT	Internet of Things
IRS	Intelligent Reflecting Surfaces
LoS	Line of Sight
ML	Machine Learning
NLoS	Non-Line of Sight
NM	Nakagami-m fading model
NOMA	Non-Orthogonal Multiple Access
OMA	Orthogonal Multiple Access
OP	Outage Probability
PDF	Probability Density Function
PE	Pointing Error
PSK	Phase Shift Keying
QAM	Quadrature Amplitude Modulation
RF	Radio Frequency Communication
RSSI	Received Signal Strength Indicator
Rx	Receiver
SAGIN	Satellite Air–Ground Integrated Network
SATCOM	Satellite Communication
SC	Switching Scheme
SEP	Symbol Error Rate Probability
SNR	Signal to Noise Ratio
SS	Soft Switching
TCP	Transmission Control Protocol
